# A pH-Sensitive Peptide-Containing Lasso Molecular Switch

**DOI:** 10.3390/molecules180911553

**Published:** 2013-09-17

**Authors:** Caroline Clavel, Karine Fournel-Marotte, Frédéric Coutrot

**Affiliations:** Supramolecular Machines and ARchitectures Team, Institut des Biomolécules Max Mousseron, (IBMM) UMR 5247 CNRS-UM1-UM2, Université Montpellier 2, Place Eugène Bataillon, Case Courrier 1706, F-34095 Montpellier Cedex 5, France

**Keywords:** [1]rotaxane, lasso, molecular switch, self-entanglement, peptide

## Abstract

The synthesis of a peptide-containing lasso molecular switch by a self-entanglement strategy is described. The interlocked [1] rotaxane molecular machine consists of a benzometaphenylene[25]crown-8 (BMP25C8) macrocycle surrounding a molecular axle. This molecular axle contains a tripeptidic sequence and two molecular stations: a *N*-benzyltriazolium and a pH-sensitive anilinium station. The tripeptide is located between the macrocycle and the triazolium station, so that its conformation can be tailored depending on the shuttling of the macrocycle from one station to the other. At acidic pH, the macrocycle resides around the anilinium moiety, whereas it shuttles around the triazolium station after deprotonation. This molecular machinery thus forces the lasso to adopt a tightened or a loosened conformation.

## 1. Introduction

As part of our research concerning the synthesis of interlocked molecular machines, we were interested in the synthesis of lasso compounds. In Nature, lasso molecules, more precisely lasso peptides, can be secreted by bacteria. These compounds contain 16 to 21 amino acid residues which define a stable lasso molecular shape (*i.e.*, a peptide macrocycle covalently linked to a peptide axle which threads the macrocycle) with a very constrained conformation, in comparison with their unthreaded analogues. The macrocycle is threaded by the peptide tail, whose bulky side-chains act as molecular barriers to trap the macrocycle around the peptide axle in the lasso conformation. The encircled peptide axle then adopts a looped conformation, conferring to the lasso a very compact tridimensional structure, which is responsible for its biological activity. Beyond their remarkable stability against proteolytic degradation, chemical and thermal denaturation [1,2,3], some lasso peptides proved to inhibit HIV replication [4] or the Gram-negative RNA polymerase [5]. As a particular example of lasso peptide, the ribosomally synthesized [6,7] antimicrobial peptide microcin J25 (MccJ25), which was isolated from *E. coli* in 1992, [8] has been extensively studied during the past years [9,10,11,12]. The amino acid sequence was first proposed by Blond *et al.* in 1999, who proposed a 21-residue, head-to-tail cyclic structure [13]. Four years later, three teams (Montelione and Ebright *et al.*, Craik *et al.*, and Darst *et al.*) demonstrated concomitantly that the initial proposed covalent and three-dimensional structure was incorrect, and that the peptide was in fact a lasso compound [14,15,16]. In view of their structural-dependent properties, lasso peptides could be considered as potential scaffold for therapeutic peptides [17]. With this aim, several biosyntheses and structure-activity analysis of a wide range of lasso peptides were realized [18,19,20]. Recently, Marahiel *et al.* have highlighted the interest of incorporating a RGD peptide sequence in the turn of the lasso MccJ25 [21]. They substituted the native Gly^12^Ile^13^Gly^14^ by the ArgGlyAsp sequence in the loop of the lasso, using site-directed mutagenesis of the precursor protein McjA. Interestingly, they found that the use of a lasso scaffold triggers a specific conformation of the incorporated bioactive peptide sequence, which is responsible for specific and distinct physical, biological and chemical properties, with respect to their linear shape. To date, no lasso peptide has been synthesized using chemical protocols, probably because of the highly difficult synthetic challenge, especially due to the folding of peptides which disturbs the necessary preorganization between the components to be assembled into an interlocked lasso. However, other more or less related works concerning peptides and interlocked molecular architectures such as peptido[2]rotaxanes have already been reported. Leigh *et al.* have been the first to surround the dipeptide unit GlyGly with a tetramide macrocycle, using hydrogen bond interactions between the peptide amide carbonyl groups of the thread and the NH of the macrocycle [22]. They next extended their efficient five-components hydrogen bond directed clipping strategy to the synthesis and the study of a wide range of dipeptides [23,24,25,26]. They also prepared many [2]rotaxane molecular shuttles in which the encircled thread contains different peptide moieties as molecular stations [27,28,29,30,31,32,33,34,35]. Inversely, some [2]rotaxanes, in which the macrocycles consist of cyclic peptides were reported too [36]. More recently, Moretto *et al.* employed the Leigh’s strategy to yield an oligopeptide [2]rotaxane shuttle, in which the longest part of the axle is a rigid helical peptide [37]. It is also noteworthy that some biological applications of the peptide rotaxanes emerged. Indeed, Leigh *et al.* prepared a pentapeptide [2]rotaxane derived from the Met-enkephalin in which the peptide core is protected against peptidase-catalyzed hydrolysis, until it is released by the action of a galactosidase [38,39]. Anderson *et al.* reported an enzymatic synthesis, using α-chymotrypsin, of a peptide rotaxane based on a cyclodextrin and a peptide thread containing a diazo moiety. They also described the resistance of the obtained peptide rotaxane against enzyme-catalyzed hydrolysis depending on the photoisomerization of the diazo moiety [40]. Meanwhile, Smithrud *et al.* designed rotaxanes as peptide carriers, using a grafted crown ether and a calix[4]arene, cyclophane, or cleft as a blocking group [41]. It remains from all these studies that protection of peptides against enzymatic degradation, on one hand, and the tailoring of the shape of peptides, on another hand, are two main problems to tackle for the conception of new efficient peptide drugs.

**Figure 1:**
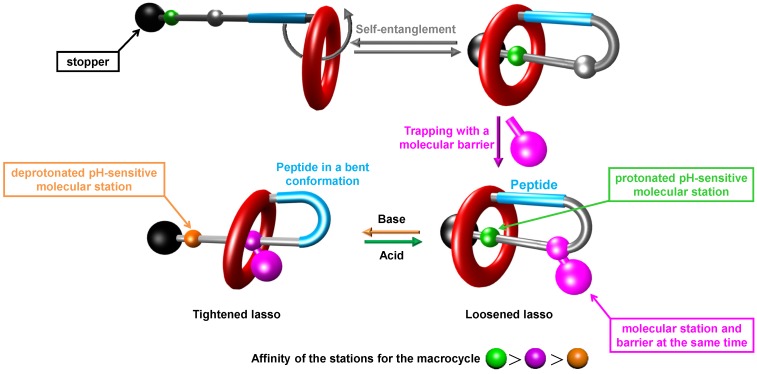
Cartoon representation of the molecular lasso targets and of the synthetic strategy used to prepare pH-sensitive peptide-containing lasso molecular switch.

Since the properties of a molecule highly rely on its topology, we were interested in combining a lasso molecular architecture that contains a peptide sequence with the molecular machinery. Therefore, we report, in this paper, a first synthetic approach of a lasso molecule containing, in its turn, the simplest GlyGlyGly peptide as a model sequence (Figure 1).

The lasso has also been designed so that it could be possible to act as a pH-sensitive molecular shuttle. Thus, the shuttling of the macrocycle along the threaded axle could trigger a variation in the conformation of the lasso (more or less tightened), hence causing a more or less bent conformation of the peptide. This concept should then be attractive for switching the properties of a peptide part of a lasso after applying an external *stimulus*.

The molecular machinery described in this paper is based on interactions between a crown ether macrocycle and our already reported system of molecular stations [42]. Indeed, the lasso compound is composed of a benzometaphenylene[25]crown-8 (BMP25C8) [43,44,45,46] macrocycle which surrounds a molecular axle containing two molecular stations for the BMP25C8. The anilinium moiety [47,48,49] is the best molecular station and is used as the molecular template for the rotaxane formation. The *N*-benzyltriazolium station is of poorer affinity, as long as the anilinium remains protonated. At the protonated state, the lasso lies in a loosened conformation without many constraints for the conformation of the peptide. However, after deprotonation, the BMP25C8 shuttles around the triazolium station, causing a tightening of the lasso and forcing the peptide to adopt a more bent conformation.

## 2. Results and Discussion

### 2.1. Synthetic Strategy to Synthesize the Lasso Compounds

The necessary driving force to yield our interlocked lasso molecules lies on the interactions between a crown ether and an ammonium moiety [50,51]. Even though the binding affinities of the molecular stations are lower for the larger macrocycle BMP25C8 than for the smaller DB24C8, the formation of the [1]rotaxane was still possible. In the present paper, the strategy used to interlock the molecular structure is based on a self-entanglement [52] of a “hermaphrodite” molecule (*i.e.*, a molecule containing both a macrocycle as host and an axle holding the anilinium template as guest) (Figure 1). The size of the cavity of the macrocycle is crucial for the successful self-entanglement strategy, especially because the end of the molecular axle is already capped with a bulky stopper, which stops the axle from threading through the macrocycle by this extremity. However, the BMP25C8 macrocycle appears large enough to allow for the rotation of the meta-substituted aromatic ring. This internal rotational movement in the BMP25C8 leads to the threading of the covalently linked anilinium-containing molecular axle, until the macrocycle surrounds the anilinium molecular template. At this stage, the lasso compound remains in equilibrium with the initial un-interlocked hermaphrodite molecule. We can then take advantage of the presence of the triazole moiety, which is located between the macrocycle and the anilinium station, in order to create the second molecular station (*i.e.*, triazolium) on one hand, and especially on the other hand, to incorporate a bulky side-chain that acts as a kinetic molecular barrier [53] and traps the interlocked architecture.

### 2.2. Preliminary Results Obtained on a Non-Peptidic Lasso Molecular Switch

**Scheme 1:**
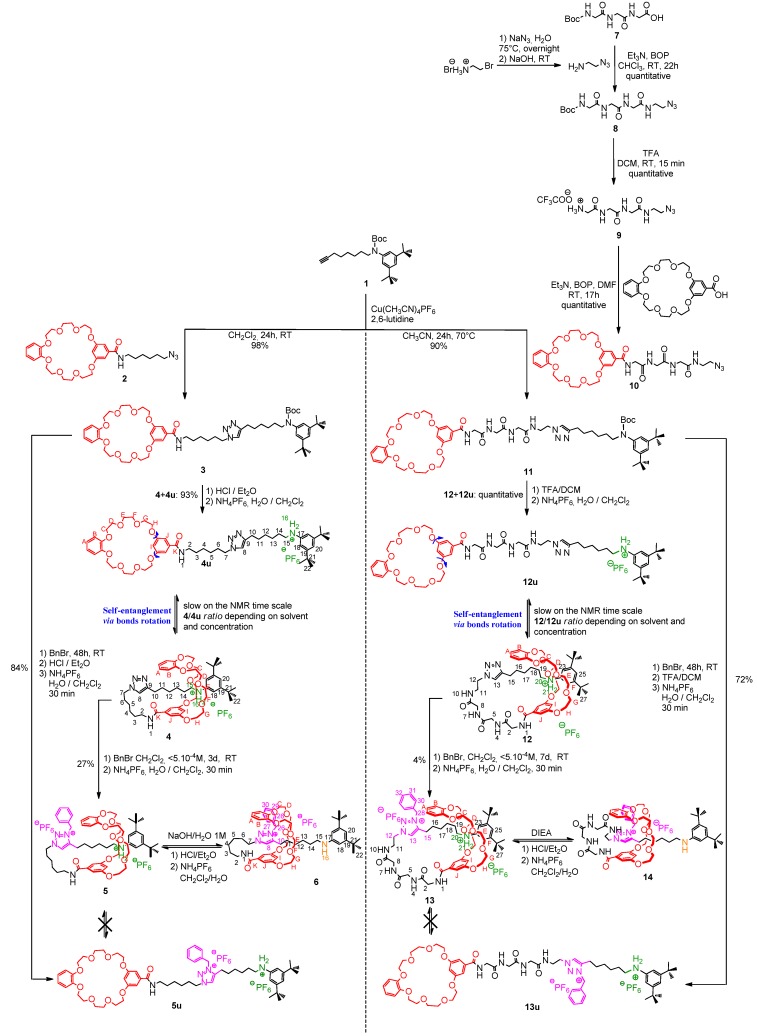
Synthesis of the molecular lassos and molecular machinery.

We have recently reported the feasibility of the self-entanglement strategy on a non-peptidic molecule [54] (Scheme 1, left side). The hermaphrodite unthreaded molecule **4u**, consisting of the BMP25C8 macrocycle linked to the anilinium molecular axle, was prepared in a two-step sequence from the already synthesized alkyne **1 [55]**. This alkyne compound possesses a carbamoylated di-*tert*-butylaniline moiety, so that no template effect can occur at this time.

The first step was a copper(I)-catalyzed Huisgen [56,57,58,59] 1,3-dipolar cycloaddition, also called “CuAAC click chemistry [60,61], between the alkyne **1** and the azido BMP25C8 **2** in dichloromethane in the presence of 2,6-lutidine and Cu(MeCN)_4_PF_6_. This step led very efficiently to the triazole compound **3**, which was then submitted to a decarbamoylation that revealed the anilinium template moiety. A slow equilibrium, on the NMR time scale, between the interlocked lasso **4** and the uncomplexed molecule **4u** could then be observed. Since the di-*tert-*butylanilinium end of the molecular axle is too bulky to thread into the macrocycle, the only way to assemble the lasso architecture is through self-entanglement. It was demonstrated that the best conditions to obtain the lasso **4** was the use of dichloromethane, as a good hydrogen bond promoting solvent, and at high dilution. In these optimal conditions, a maximum *ratio* **4**/**4u** of 45/55 was measured. Subsequent trapping of the lasso structure was realized by taking advantage of the localization of the triazole moiety between the macrocycle and the anilinium molecular station. The benzylation of the triazole afforded the lasso **5** in a 27% yield. Here, no more equilibrium between interlocked compound **5** and uncomplexed compound **5u** was possible, due to the incorporation of the benzyl moiety on the triazole which acts as a steric molecular barrier for the BMP25C8. After deprotonation of the lasso **5**, we found that the BMP25C8 shuttles toward the triazolium molecular station, thus tightening the lasso structure. This process could be reversed in acidic medium.

### 2.3. Extension to the Preparation of a pH-Sensitive Tripeptide-Containing Lasso Molecular Switch

#### 2.3.1. Synthetic Part

We then aimed to incorporate a peptide sequence in the turn of the lasso molecular architecture. The simplest GlyGlyGly tripeptidic sequence was chosen in order to fit with the structural prerequisites for the self-entanglement synthetic strategy. Indeed, no such self-entanglement should be authorized with bulky amino acid side-chains. With the aim to be able to tailor the conformation of the peptide depending on the molecular machinery applied to the lasso, the peptide sequence has been localized the closest to the macrocycle BMP25C8, that is to say in the loop of the lasso. Thus, the azido-containing macrocycle **10** was first synthesized according to a three-step sequence from the commercially available BocGlyGlyGlyOH. Activation of the *C*-terminal side of the tripeptide using Castro’s reagent BOP [62] in the presence of triethylamine and 2-azidoethylamine [63] afforded the azido tripeptide **8** in quantitative yield. The further decarbamoylation using trifluoroacetic acid provided the ammonium intermediate **9**, which was then submitted to a coupling reaction with the carboxylic acid substituted BMP25C8 to yield the azido macrocycle **10**. Due to the very poor solubility of compound **10** in dichloromethane and in acetonitrile at room temperature, the copper(I)-catalyzed Huisgen 1,3-dipolar cycloaddition was accomplished with alkyne **1** in acetonitrile and at a temperature of 70 °C. In these experimental conditions, 90% of the unthreaded compound **11** was isolated. Decarbamoylation of **11** unmasked the anilinium template and allowed for the self-entanglement of the hermaphrodite molecule **12u**. The equilibrium between the lasso **12** and its unthreaded analogue **12u** was studied in details (see Section 2.3.2.). The subsequent benzylation of the lasso **12** allowed for the trapping of the lasso molecular architecture: it was carried out in dichloromethane at high dilution with respect to **12** and using a very large excess of benzyl bromide. Although the conversion rate for the benzylation of the non-peptidic lasso **4** was quasi quantitative (only 5% of the starting triazole compounds **4**/**4u** were recovered after chromatographic columns), the formation of the benzyl triazolium appears much more tricky in the case of the peptide-containing lasso. Indeed, after 7 days, 33% of the triazole compounds **12**/**12u** were still detected by NMR spectroscopy. Moreover, several chromatographic columns on both silica gel and sephadex yielded to a mixture composed of 17% of the unthreaded benzyltriazolium **13u** and 17% of the desired triazolium lasso **13**. The separation between these compounds was found to be very tough and only 4% of **13** could be isolated. We were curious about the possibility to realize the benzylation at a higher temperature in order to accelerate the rate of the reaction. Unfortunately, VT ^1^H-NMR experiments in C_2_D_2_Cl_4_ on **12**/**12u** showed that increasing the temperature results in the dramatic decrease of the proportion of the lasso compound **12** (Table 1, entries 3–7). Despite the poorly efficient reaction of benzylation, the deprotonation of the isolated loosened lasso **13** was carried out efficiently by adding the Hünig’s [64] base DIEA. Deprotonation of the anilinium triggered the shuttling of the BMP25C8 towards the triazolium moiety, hence causing the tightening of the lasso and inducing the more constraint conformation of the tripeptide (see Section 2.3.3.).

#### 2.3.2. Studies of the Equilibrium between the Unthreaded Compound **12u** and the Lasso **12**

**Figure 2:**
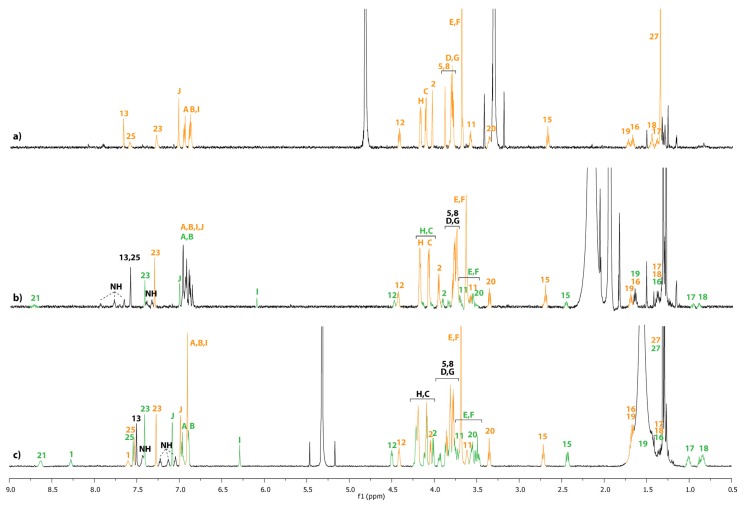
^1^H-NMR spectra (600 MHz, 298 K) at 5.10^−4^ M of a mixture of compounds **12/12u** in: (**a**) CD_3_OD. (**b**) CD_3_CN. (**c**) CD_2_Cl_2_. The lettering and numbering correspond to the proton assignments indicated in scheme 1. The orange color corresponds to the unthreaded molecule **12u**, whereas the green color corresponds to lasso compound **12**. The black assignments correspond to both compounds.

**Figure 3:**
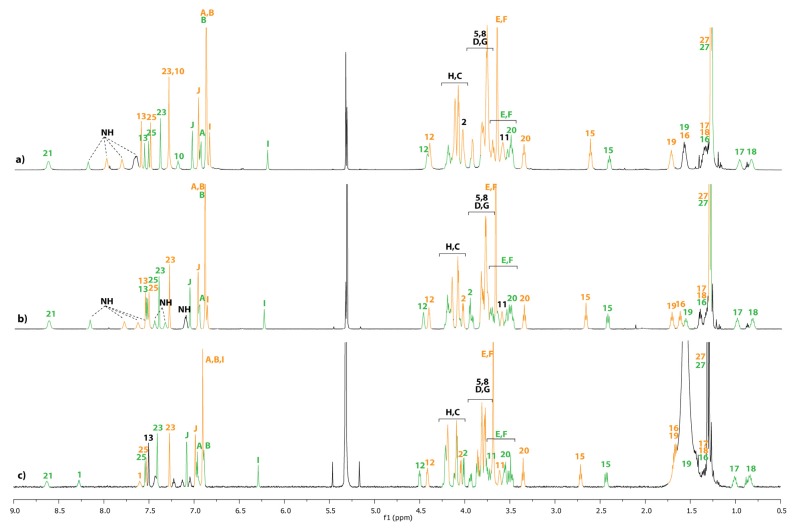
^1^H-NMR spectra (600 MHz, 298 K) of a mixture of compound **12/12u** in CD_2_Cl_2_ at: (**a**) 5.10^−2^ M. (**b**) 5.10^−3^ M. (**c**) 5.10^−4^ M. The lettering and numbering correspond to the proton assignments indicated in scheme 1. The orange color corresponds to the unthreaded molecule **12u**, whereas the green color corresponds to lasso compound **12**.

The equilibrium between the unthreaded compound **12u** and the lasso **12** highly depends on the polarity of the solvent (Figure 2), on the concentration (Figure 3), and on the temperature.

Table 1 summarizes the **12**/**12u** ratios measured in the different solvents from the best hydrogen bond promoting solvents dichloromethane and tetrachloroethane to the more polar solvent methanol and upon variation of the concentration and temperature. As the equilibrium between **12** and **12u** proved to be slow on the NMR time scale, two sets of ^1^H-NMR signals were observed for each unthreaded and lasso compounds. The ratio values reported in Table 1 were obtained by ^1^H- NMR spectroscopy at 600 MHz by integrating the signal of hydrogens H_15_ or H_12_.

**Table 1:** Ratio between lasso compound **12** and unthreaded molecule **12u** depending on concentration, solvent and temperature.

Entries	*C* (12/12u) [M]	T (K)	CD_3_OD	CD_3_CN	CD_2_Cl_2_	C_2_D_2_Cl_4_
1	5.10^−2^	298	-	-	35/65	-
2	5.10^−3^	298	-	-	44/56	-
3	5.10^−4^	298	0/100	25/75	47/53	55/45
4	5.10^−4^	318	-	-	-	38/61
5	5.10^−4^	328	-	-	-	29/71
6	5.10^−4^	338	-	-	-	14/86
7	5.10^−4^	348	-	-	-	8/92

Obviously, it can be seen from the data reported in Table 1 that the nature of the solvent has a big influence on the **12**/**12u** ratio. At 298 K, the best self-entanglement was noticed in the less polar dichloromethane and tetrachloroethane and at the highest dilution (Table 1, entry 3). At a similar concentration, the proportion of the lasso **12** dramatically declines in the more polar acetonitrile and no presence of the lasso was detected in methanol (Figure 2 and Table 1, entry 3). Eventually, increasing the concentration of the sample in dichloromethane tends to be unfavorable to the interlocked lasso architecture (Figure 3 and Table 1, entries 1–3).

**Figure 4:**
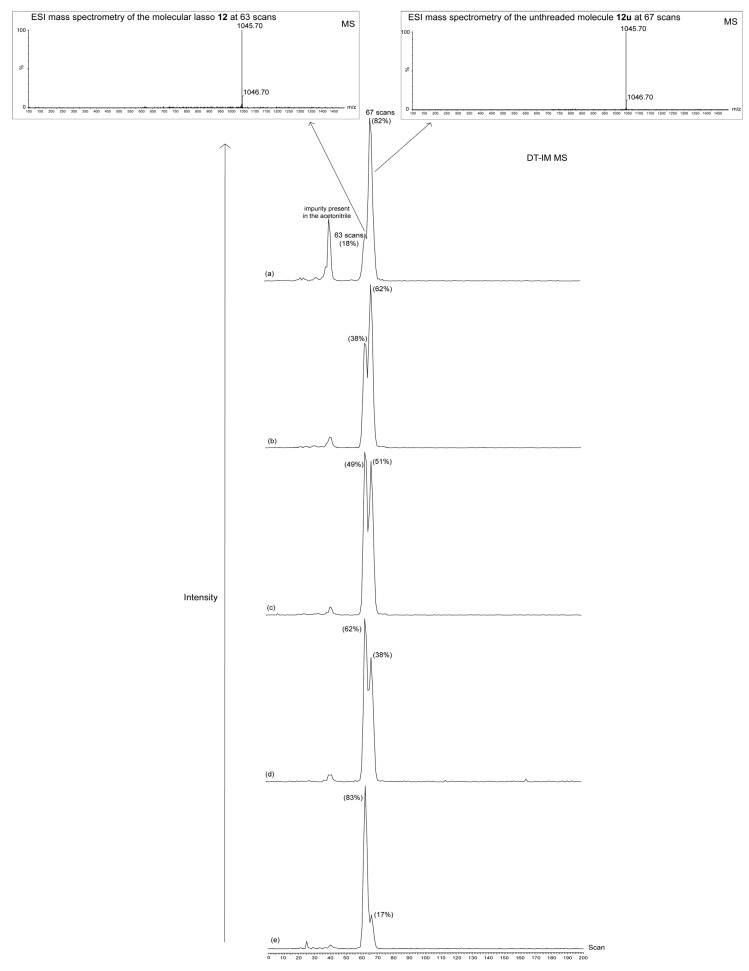
DT IM-MS of the mixture **12**/**12u** in: (**a**) CH_3_CN. (**b**) CH_3_CN/CH_2_Cl_2_ 30/70. (**c**) CH_3_CN/CH_2_Cl_2_ 20/80. (**d**) CH_3_CN/CH_2_Cl_2_ 10/90. (**e**) CH_2_Cl_2_. Data shows: the average time distribution (in scan units) at a drift voltage of 40V and the peak area (in %) for the detected molecular ion [M−PF_6_]^+^.

Besides, the equilibrium between **12u** and **12** could be highlighted using drift tube ion mobility mass spectrometry (DT IM-MS). By this analytical method, it was possible to separate compounds **12** and **12u**, because they have different velocities which are due to their distinct sizes related to their unlike conformational structures. A similar analytical study was utilized by Leigh *et al.* to distinguish a trefoil knot from its unknotted-macrocycle analogue [65]. Figure 4 shows the DT IM-MS results of various samples of a mixture of compounds **12**/**12u** at the same concentration (5 × 10^−4^ M) upon variation of the ratio of a solvent mixture of dichloromethane/acetonitrile. Interestingly, and whatever the polarity of the solvent mixture used for the injection, the lasso compound **12**, whose conformation is smaller and more compact, have a lower arrival time distribution (63 scans). On the contrary, the unthreaded molecule **12u** consists of a larger ion with less conformational restrictions. As a consequence, it takes more time for this ion to travel through the drift cell (arrival time distribution of 67 scans). Moreover, the same trend in the **12**/**12u** ratio could be detected upon variation of the solvent polarity by this method as that observed by NMR. Indeed, a **12**/**12u** ratio of 18/82 in favor of **12u** was measured in the more polar acetonitrile, whereas the ratio was inverted in pure dichloromethane. The consistency of this trend observed from the dichloromethane to the acetonitrile, with respect to the NMR study, suggests that the equilibrium between **12** and **12u** is slow on the ion mobility mass spectrometry time scale. The width at half-height of each pick was also measured in pure acetonitrile and dichloromethane: the peak related to the lasso **12** has a slightly narrowest distribution, which reveals a more persistent size and shape, in accordance with the more restraint lasso structure. On the contrary, the slightly broadest distribution observed for the peak of the unthreaded molecule **12u** exhibits its larger degree of flexibility.

#### 2.3.3. ^1^H-NMR Investigation of the Molecular Machinery between Lasso Compounds **13** and **14**

**Figure 5:**
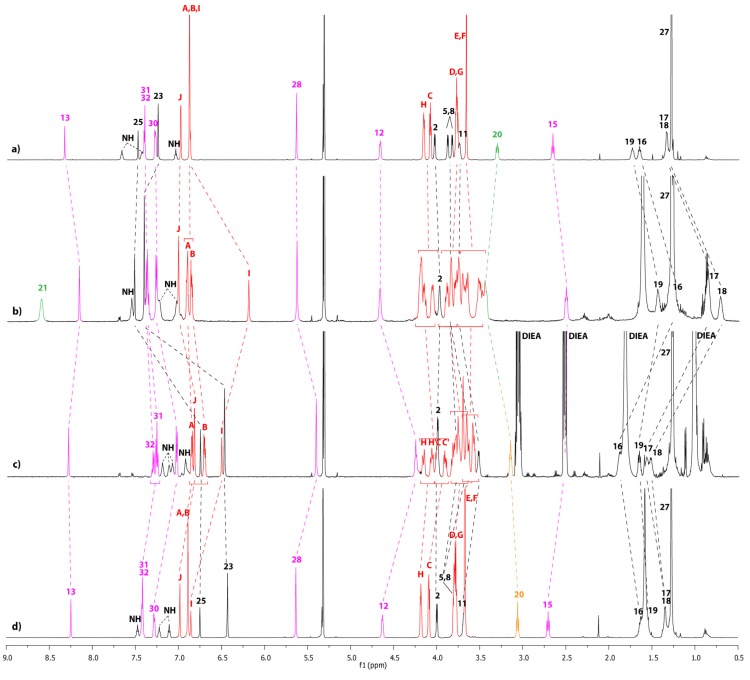
^1^H-NMR spectra (600 MHz, CD_2_Cl_2_, 298 K) of: (**a**) the protonated unthreaded compound **13u**. (**b**) the protonated lasso-based compound **13**. (**c**) the deprotonated lasso-based compound **14**. (**d**) the deprotonated unthreaded compound **14u**. The coloring, lettering and numbering correspond to the proton assignments indicated in scheme 1.

The comparison between the ^1^H-NMR spectra of the protonated and deprotonated unthreaded molecules **13u**, **14u** with their respective protonated and deprotonated molecular lassos **13** and **14** allowed to demonstrate the lasso interlocked architecture and the localization of the BMP25C8 around the molecular axle (Figure 5).

In the ^1^H-NMR spectrum of the protonated molecular lasso **13**, signals for the hydrogen atoms H_A-B_ and H_C-F_ of the BMP25C8 appear split because they are facing the two non-symmetrical ends of the molecular axle of the interlocked lasso architecture. Moreover, the localization of the macrocycle around the anilinium station in **13** can be deduced from the direct comparison between the ^1^H-NMR spectra of **13** and **13u** [Figure 5(a) and (b)]. Indeed, in the lasso **13**, the signals for the hydrogen atoms H_21_appear at very high chemical shift (δ = 8.60 ppm). Likewise, hydrogens H_20_ belonging to the anilinium station are shifted downfield with respect to the protonated unthreaded molecule **13u** (Δδ = 0.15 ppm), because they interact by hydrogen bonds with the oxygen atoms of the BMP25C8. Concerning the hydrogen H_I_ of the BMP25C8, it is dramatically shifted upfield in **13** (Δδ = −0.67 ppm) because it experiences the shielding effect of the anilinium aromatic ring. The same trend is observed for hydrogen atoms H_E-F_ of the BMP25C8. Eventually, the hydrogen atoms H_13_ and H_15–19_ are all more or less shielded (respectively Δδ = −0.17, −0.15, −0.40, −0.50, −0.63 and −0.30 ppm) because they all undergo the shielding effect of the aromatic rings of the BMP25C8. Among them, it is noteworthy that hydrogen atoms H_16-17-18_ are the more concerned by this effect.

Deprotonation of the loosened lasso **13** led to the tightened lasso **14**. The direct comparison of the ^1^H-NMR spectra between the two lasso [1]rotaxanes **13** and **14** reveals the shuttling of the BMP25C8 [Figure 5(b) and (c)]. Unsurprisingly, ^1^H-NMR chemical shifts of hydrogens H_20_, H_23_ and H_25_, which belong to the anilinium moiety, are shifted upfield in the deprotonated lasso **14**, due to the deprotonation of the anilinium unit. At the same time, in **14**, H_I_ is shifted downfield (Δδ = 0.31 ppm) because it does not experience anymore the strong shielding effect of the aniline extremity. However, this signal is still at a lower chemical shift than those observed in the unthreaded protonated and deprotonated molecules **13u** and **14u** (respectively Δδ = −0.36 and −0.34 ppm) [Figure 5(c) and (d)]. This result can be explained by the new localization of H_I_ which now experiences the shielding effect of the benzyl triazolium moiety, this latter effect being weaker than those of the anilinium aromatic ring. This observation is corroborated by the upfield shift underwent by the other hydrogen atoms of the aromatic rings of the crown ether H_J_, H_A_ and H_B_ (respectively Δδ = −0.18, −0.04 and −0.16 ppm), which experience the same shielding effect from the triazolium ring. Moreover, the displacement of the BMP25C8 can account for the upfield shift observed in **14** for the hydrogen atoms of the benzyl molecular barrier H_28_, H_30_ (respectively Δδ = −0.23, −0.24 ppm) and in a lesser extent H_32_ (with respect to **13**), due to their localization in the shielding cavity of the aromatic rings of the BMP25C8. Similarly, the hydrogen atoms of the molecular axle located on the other side of the molecular barrier H_11_ and H_12_ are both shielded by the BMP25C8: actually, this latter lies on the triazolium moiety and its aromatic rings can reach the other side of the molecular barrier (respectively Δδ = −0.23, −0.41 ppm). On the contrary, the hydrogen atoms located between the aniline and the triazolium unit H_16_, H_17_, H_18_ and H_19_ and in a much lesser extent H_13_, are shifted downfield in **14** (respectively Δδ = 0.62, 0.73, 0.82, 0.22 and 0.13 ppm) because they do not experience anymore the shielding effect of the BMP25C8. The direct comparison between the ^1^H-NMR spectra of the tightened deprotonated lasso **14** and the deprotonated unthreaded analogue **14u** corroborates the localization of the BMP25C8 [Figure 5(c) and (d)].

**Figure 6:**
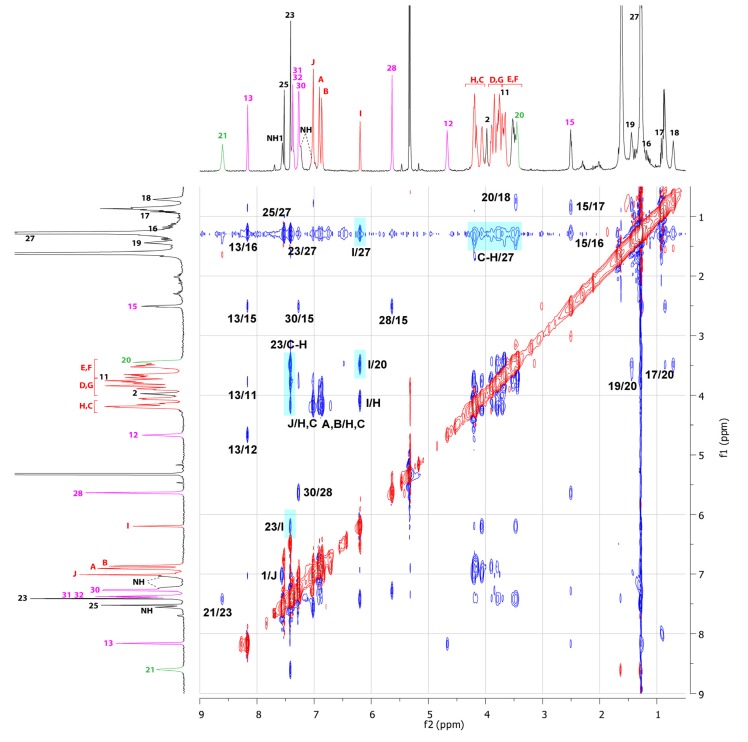
^1^H-NMR ROESY experiment (600 MHz, CD_2_Cl_2_, 298 K) of the protonated loosened molecular lasso **13**. The coloring, lettering and numbering correspond to the proton assignments indicated in Scheme 1.

**Figure 7:**
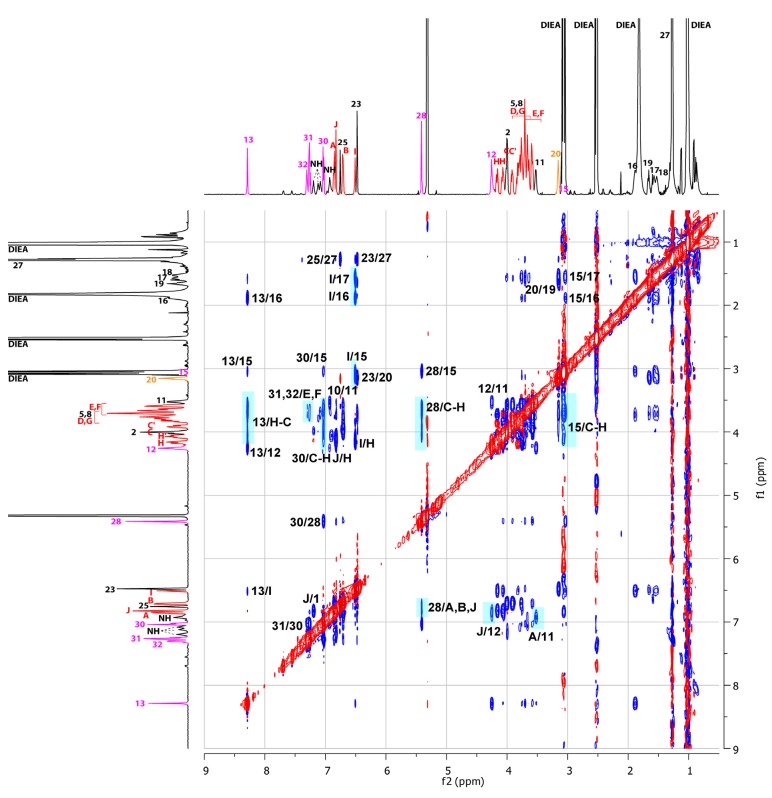
^1^H-NMR ROESY experiment (600 MHz, CD_2_Cl_2_, 298 K) of the deprotonated tightened molecular lasso **14**. The coloring, lettering and numbering correspond to the proton assignments indicated in Scheme 1.

The ROESY ^1^H-NMR experiments provided complementary and consistent insights into the structure of the lasso molecular switch **13**/**14** (Figure 6 and Figure 7). The ROESY ^1^H-NMR experiment carried out on the protonated lasso (Figure 6) provided the evidences of the lasso architecture with a localization of the BMP25C8 around the anilinium station (relevant correlation peaks highlighted in cyan). It is particularly true for the correlation peaks between respectively H_23_ and H_27_ of the anilinium site with H_C-H_ of the BMP25C8, or between H_I_ of the BMP25C8 with H_20_, H_23_ and H_27_. Interestingly, no correlation peak between the BMP25C8 and the triazolium site was observed, which is consistent with the localization of the macrocycle around the anilinium moiety.

After deprotonation (Figure 7), the relevant highlighted cross peaks relative to the loosened lasso disappear, whereas new correlation peaks demonstrate the new main localization of the macrocycle around the triazolium. In particular, new correlation peaks are observed for the hydrogens H_C-H_ belonging to the BMP25C8 with respectively the triazolium hydrogen H_13_, the benzyl barrier H_28_ and H_30_, and the next hydrogen H_15_. At the same time, hydrogen atoms H_E-F_ correlate with H_31-32_ of the aromatic benzyl molecular barrier. The aromatic hydrogen H_I_ of the BMP25C8 is now correlated with the hydrogen atoms H_15_, H_16_ and H_17_, whereas the other aromatic hydrogens H_A_ and H_B_ of the BMP25C8 are correlated with the benzyl barrier H_28_. In a consistent way, the aromatic hydrogen H_J_ of the BMP25C8 is correlated with H_12_, whereas the last aromatic hydrogen of the BMP25C8 H_A_ is now correlated with H_11_. The whole NMR studies are consistent with the two main localizations of the BMP25C8 around either the anilinium or the triazolium station upon variation in pH. 

Concerning the peptide part GlyGlyGly of the lasso molecule, the overlapping in ROESY spectra between methylene hydrogens H_2_, H_5_, H_8_ and the very split methylene hydrogens H_C-H_ of the BMP25C8 unfortunately prevents us from giving dependable assumptions concerning an eventual conformational change of the tripeptide sequence. However, slight changes in chemical shifts could be observed when comparing the 1D ^1^H-NMR spectra of the protonated loosened lasso **13** with the deprotonated tightened lasso **14**. The ^1^H-NMR chemical shift of methylene hydrogens H_2_ remained almost unchanged in **14** (Δδ = +0.02 ppm), whereas H_5_ and H_8_ are more or less shielded in **14 **(Δδ = −0.05 and −0.18 ppm). This latter observation can be assigned to the shielding effect of the aromatic of the BMP25C8, corroborating the new localization of the BMP25C8.

## 3. Experimental

### 3.1. General

All reactions were achieved under an atmosphere of argon unless otherwise indicated. Dichloromethane was distilled over P_2_O_5_ and was degassed by bubbling Ar for 20 min. Analytical thin-layer chromatography (TLC) was performed on Merck silicagel 60 F254 plates. Compounds were visualized by dipping the plates in an ethanolic solution of ninhydrine, followed by heating. ^1^H-NMR and ^13^C-NMR spectra were obtained from a Bruker Avance III spectrometers (respectively at 400.13 MHz or 600.13 MHz and 100.62 MHz or 150.95 MHz). Chemical shifts of ^1^H-NMR and ^13^C-NMR are given in ppm by using CHD_2_OH or CHDCl_2_ as references (3.31 ppm and 5.32 ppm respectively for ^1^H spectrum and 49 ppm and 54 ppm respectively for ^13^C spectrum). Coupling constants (*J*) are reported in hertz (Hz). Standard abbreviations indicating multiplicity were used as follows: s (singlet), br (broad), d (doublet), t (triplet), q (quartet), m (multiplet). High-resolution mass spectra (HRMS) and mass spectra were recorded on a Q-TOF Micro (water) apparatus.

### 3.2. Synthesis and Characterizations of [1]Rotaxanes **13** and **14**

*Boc-GlyGlyGly-NH-CH_2_CH_2_-N_3_* (**8**). 2-Azidoethylamine was first prepared by adding sodium azide (800 mg, 12.2 mmol, 2.3 equiv) to a solution of 2-bromoethylamine hydrobromide (1.08 g, 5.3 mmol, 1 equiv) in H_2_O (20 mL). The resulting solution was then stirred overnight at 65 °C. After cooling down to RT, NaOH (232 mg, 5.8 mmol, 1.1 equiv) was added to the reaction mixture. The resulting aqueous solution was saturated with NaCl and extracted with CHCl_3_ (3 × 20 mL). The organic layer was dried over anhydrous MgSO_4_ and used directly for the next step without being concentrated. The solution containing the 2-azidoethylamine (5.3 mmol, 1.5 equiv) was diluted with CHCl_3_ (total volume: 150 mL) before adding successively Boc-glycylglycylglycine (1 g, 3.5 mmol, 1 equiv) and triethylamine (1.43 mL, 10.6 mmol, 3 equiv). The mixture was then stirred until complete dissolution of the tripeptide (15 min). BOP (1.88 g, 4.6 mmol, 1.3 equiv) was added and the mixture was stirred at RT for 18 h. The resulting white precipitate **8** was filtered, washed with CH_2_Cl_2_ and dried under vacuum. It was used in the next step without further purification (1.25g, quantitative). R_f_: 0.40 (CH_2_Cl_2_/MeOH 9/1). ^1^H-NMR (600 MHz, CD_3_OD, 298K): δ ppm = 3.90 & 3.86 & 3.75 (3s, 3x2H, H_2_ H_5_ H_8_), 3.45–3.34 (m, 4H, H_11_ H_12_), 1.45 (s, 9H, H_CH3-Boc_). ^13^C-NMR (150 MHz, CD_3_OD, 298K): δ ppm = 173.6 & 172.2 & 171.9 (C_3_ C_6_ C_9_), 158.8 (CO_-Boc_), 81.0 (C(CH_3_)_3-Boc_), 51.3 (C_12_), 44.9 & 43.8 & 43.4 (C_2_ C_5_ C_8_), 39.9 (C_11_), 28.7 (CH_3-Boc_). MS (ESI): [M + H]^+^ calcd for C_13_H_24_N_7_O_5_: 358.18, found: 358.19.

*TFA,H-GlyGlyGly-NH-CH_2_CH_2_-N_3_* (**9**). TFA (5 mL) was added to a suspension of the Boc-GlyGlyGly-NHCH_2_CH_2_N_3_ **8** (1.25g) in CH_2_Cl_2_ (20 mL), leading to complete dissolution of this compound. After stirring for 15 min at RT, the reaction mixture was concentrated and co-evaporated 5 times with CH_2_Cl_2_ (20 mL) in order to remove the excess of TFA. The resulting white solid **9** (1.15 g, quantitative) could be used without further purification. R_f_: 0.09 (CH_2_Cl_2_/MeOH 9/1). ^1^H-NMR (600 MHz, CD_3_OD, 298K): δ ppm = 3.98 & 3.88 & 3.78 (3s, 3 × 2H, H_2_ H_5_ H_8_), 3.39 (s, 4H, H_11_ H_12_). ^13^C-NMR (150 MHz, CD_3_OD, 298K): δ ppm = 171.9 & 171.8 & 168.4 (C_3_ C_6_ C_9_), 161.0 (q, ^2^*J*_C-F_ = 38.3 Hz, CO_2-TFA_), 117.1 (q, ^1^*J*_C-F_ = 287.6 Hz, CF_3-TFA_), 51.4 (C_12_), 43.6 & 43.4 & 41.6 (C_2_ C_5_ C_8_), 39.9 (C_11_). MS (ESI): [M−TFA]^+^ calcd for C_8_H_16_N_7_O_5_: 258.13, found: 258.13.

*Azido macrocycle* (**10**). The preliminary synthesized carboxylic acid macrocycle [8] (894 mg, 1.82 mmol, 1 equiv) and compound **9** (707 mg, 2 mmol, 1.1 equiv) were dissolved in DMF (20 mL). Then, triethylamine (755 µL, 5.45 mmol, 3 equiv) and BOP (973 mg, 2.36 mmol, 1.3 equiv) were added and the mixture was stirred at RT for 17h. DMF was evaporated under *vacuum* and the resulting yellow oil was diluted with CH_2_Cl_2_ (100 mL), before being successively washed with an aqueous solution of HCl 1M (100 mL), a saturated aqueous solution of NaHCO_3_ (100 mL) and brine (100 mL). The organic layer was then dried over MgSO_4_ and concentrated to yield a beige precipitate, which was then triturated, washed with Et_2_O (200 mL) and dried under vacuum. The compound **10** was pure enough to be used without further purification (1.33g, quantitative). R_f_: 0.63(CH_2_Cl_2_/MeOH 9/1). ^1^H-NMR (600 MHz, CD_3_OD, 298K): δ ppm = 7.04 (d, 2H, ^4^*J*_HJ-HI_ = 2.1 Hz, H_J_), 6.98–6.92 (m, 2H, H_A_), 6.91–6.85 (m, 3H, H_I_ H_B_), 4.22–4.16 (m, 4H, H_H_), 4.14–4.08 (m, 4H, H_C_), 4.04 (s, 2H, H_2_), 3.91 & 3.89 (2s, 2 × 2H, H_5_ H_8_), 3.84–3.77 (m, 8H, H_D_ H_G_), 3.69 (s, 8H, H_E_ H_F_), 3.35–3.32 (2m, 2x2H, H_11_ H_12_). ^13^C-NMR (150 MHz, CD_3_OD, 298K): δ ppm = 172.9 & 172.2 & 171.9 & 170.4 (C_K_ C_3_ C_6_ C_9_), 161.6 & 150.5 & 136.6 (C_IV arom BMP25C8_), 123.0 (C_B_), 116.8 (C_A_), 108.0 (C_J_), 107.5 (C_I_), 72.0 & 71.9 (C_E_ C_F_), 71.0 (2s, C_D_ C_G_), 70.3 (C_C_), 69.4 (C_H_), 51.3 (C_12_), 44.5 (C_2_), 44.0 & 43.4 (C_5_ C_8_), 39.9 (C_11_). MS (ESI): [M + H]^+^ calcd for C_33_H_46_N_7_O_12_: 732.32, found: 732.32.

*Compound* (**11**). To a solution of the azido macrocycle **10** (165 mg, 0.225 mmol, 1 equiv) and the preliminary synthesized alkyne **1** [9] (103 mg, 0.25 mmol, 1.1 equiv) in dry CH_3_CN (8 mL) at 70 °C, were added successively Cu(CH_3_CN)_4_PF_6_ (84 mg, 0.225 mmol, 1 equiv) and 2,6-lutidine (3 μL, 0.03 mmol, 0.1 equiv). The mixture was stirred for 24 h at 70 °C. After filtration, the solvent was evaporated under vacuum. The crude was then purified by chromatography on a silicagel column (solvent gradient elution CH_2_Cl_2_ to CH_2_Cl_2_/MeOH 80/20) to give compound **11** (234 mg, 90%) as a beige powder. R*_f_* 0.65 (CH_2_Cl_2_/MeOH 9/1). ^1^H-NMR (600 MHz, CD_2_Cl_2_, 298K): δ (ppm) = 7.99 (br t, 1H, H_1_), 7.70 & 7.46 (2 br t, 2 × 1H, H_4_ H_7_), 7.36 (s, 1H, H_13_), 7.26 (s, 1H, H_25_), 7.11 (br t, 1H, H_10_), 7.01–6.95 (m, 4H, H_23_ H_J_), 6.92–6.86 (m, 4H, H_A_ H_B_), 6.83 (s, 1H, H_I_), 4.32 (t, 2H, ^3^*J_H12-H11_* = 5.3 Hz, H_12_), 4.18–4.13 (m, 4H, H_H_), 4.12–4.07 (m, 4H, H_C_), 4.00 (d, 2H, ^3^J*_H2-H1_* = 5.1 Hz, H_2_), 3.86–3.74 (m, 12H, H_5_ H_8_ H_D_ H_G_), 3.67 (s, 8H, H_E_ H_F_), 3.59–3.51 (m, 4H, H_11_ H_20_), 2.61 (t, 2H, ^3^*J_H15-H16_* = 7.4 Hz, H_15_), 1.64–1.56 (m, 2H, H_16_), 1.56–1.48 (m, 2H, H_19_), 1.39 (s, 9H, H_CH3-Boc_), 1.31 (s, 18H, H_27_), 1.36–1.25 (m, 4H, H_17_ H_18_). ^13^C-NMR (150 MHz, CD_2_Cl_2_, 298K): δ (ppm) = 171.8 & 170.6 & 170.4 & 168.7 (C_K_ C_3_ C_6_ C_9_), 160.7 & 149.4 & 135.8 (C_IV arom BMP25C8_), 155.6 (CO_-Boc_), 151.7 (C_24_), 142.7 (C_22_), 122.3 (C_13_), 122.2 (C_B_), 121.9 (C_23_), 120.3 (C_25_), 115.4 (C_A_), 107.5 (C_J_), 106.5 (C_I_), 80.0 (C(CH_3_)_3-Boc_), 71.3 (2s, C_E_ C_F_), 70.3 & 70.2 (C_D_ C_G_), 69.3 (C_C_), 68.9 (C_H_), 50.7 (C_20_), 49.7 (C_12_), 44.7 (C_2_), 43.9 & 43.5 (C_5_ C_8_), 39.8 (C_11_), 35.3 (C_26_), 31.7 (C_27_), 29.9 (C_16_), 28.9 (C_19_), 28.7 (CH_3-Boc_), 29.5 & 27.1 (C_17_ C_18_), 26.0 (C_15_). MS (ESI): [M + H]^+^ calcd for C_60_H_89_N_8_O_14_: 1145.65, found: 1145.65.

*Compounds* (**12/12u**). TFA (2*.*5 mL) was added to a solution of compound **11** (156 mg, 0.14 mmol, 1 equiv.) in CH_2_Cl_2_ (10 mL). After stirring for 30 min at RT, the reaction mixture was concentrated and co-evaporated 5 times with CH_2_Cl_2_ (20 mL) in order to remove the excess of TFA. The residue was then diluted in CH_2_Cl_2_ (10 mL). To this solution was added NH_4_PF_6_ (114 mg, 0.7 mmol, 5 equiv) and H_2_O milliQ (5 mL): the biphasic solution was stirred vigorously for 30 minutes. The aqueous layer was then extracted with CH_2_Cl_2_ (3 × 5 mL) and the combined organic layers were dried over MgSO_4_ and concentrated to give compounds **12**/**12u** (162 mg, quantitative) as a beige powder. R*_f_* 0.52 (CH_2_Cl_2_/MeOH 9/1). HRMS (ESI): [M+H−PF_6_]^2+^ calcd for C_55_H_82_N_8_O_12_: 523.3026, found: 523.3007.

*Unthreaded Compound* (**12u**)***.***^1^H-NMR (600 MHz, 5.10^−2^ M in CD_2_Cl_2_, 298K): δ (ppm) = 7.98 (br t, 1H, H_1_), 7.81 (br t, 1H, H_4_ or H_7_), 7.69–7.62 (m, 1H, H_4_ or H_7_), 7.60 (s, 1H, H_13_), 7.50 (t, 1H, ^4^*J_H25-H23_* = 0.9 Hz, H_25_), 7.31–7.26 (m, 1H, H_10_), 7.29 (d, 2H, ^4^*J_H23-H25_* = 0.9 Hz, H_23_), 6.97 (d, 2H, ^4^*J_HJ-HI_* = 1.7 Hz, H_J_), 6.83 (s, 4H, H_A_ H_B_), 6.84 (br t, 1H, H_I_), 4.40 (br t, 2H, H_12_), 4.14–4.10 (m, 4H, H_H_), 4.10–4.07 (m, 4H, H_C_), 4.05–4.02 (m, 2H, H_2_), 3.85–3.74 (m, 12H, H_5_ H_8_ H_D_ H_G_), 3.65 (s, 8H, H_E_ H_F_), 3.62–3.56 (m, 2H, H_11_), 3.36 (t, 2H, ^3^*J_H20-H19_* = 7.3 Hz, H_20_), 2.62 (t, 2H, ^3^*J_H15-H16_* = 6.9 Hz, H_15_), 1.76–1.69 (m, 2H, H_19_), 1.63–1.54 (m, 2H, H_16_), 1.41–1.32 (m, 2H, H_18_), 1.35–1.22 (m, 2H, H_17_), 1.29 (s, 18H, H_27_). ^13^C-NMR (150 MHz, 5.10^−2^ M in CD_2_Cl_2_, 298K): δ (ppm) = 172.0 & 170.8 & 169.2 (4s, C_K_ C_3_ C_6_ C_9_), 160.8 & 149.1 & 135.5 (C_IV arom BMP25C8_), 154.3 (C_24_), 147.5 (C_22_), 124.3 (C_25_), 124.0 (C_13_), 122.3 (C_A_), 117.3 (C_23_), 115.3 (C_B_), 107.5 (C_J_), 107.1 (C_I_), 71.1 (C_E_ C_F_), 70.4 & 70.0 (C_D_ C_G_), 69.2 (C_C_), 68.9 (C_H_), 53.9 (C_20_), 50.3 (C_12_), 44.6 (C_2_), 43.9 & 43.4 (C_5_ C_8_), 39.9 (C_11_), 35.6 (C_26_), 31.5 (C_27_), 28.7 & 28.0 (C_16_ C_17_), 25.9 (C_18_), 25.8 (C_19_), 24.9 (C_15_). 

*Pseudo*[1]*rotaxane* (**12**)***.***^1^H-NMR (600 MHz, 5.10^−2^ M in CD_2_Cl_2_, 298K): δ (ppm) = 8.63 (br s, 2H, H_21_), 8.19 (br t, 1H, H_1_), 7.69–7.62 (m, 2H, H_4_ H_7_), 7.56 (s, 1H, H_13_), 7.52 (br t, 1H, H_25_), 7.39 (d, 2H, ^4^*J_H23-H25_*= 1.2 Hz, H_23_), 7.19 (t, 1H, ^3^*J_H10-H11_* = 5.8 Hz, H_10_), 7.03 (d, 2H, ^4^*J_HJ-HI_* = 1.7 Hz, H_J_), 6.96–6.92 (m, 2H, H_A_), 6.90–6.86 (m, 2H, H_B_), 6.20 (br t, 1H, H_I_), 4.43 (br t, 2H, H_12_), 4.24–3.99 (m, 8H, H_H_ H_C_), 3.94–3.91 (m, 2H, H_2_), 3.96–3.67 (m, 12H, H_5_ H_8_ H_D_ H_G_), 3.73–3.46 (m, 8H, H_E_ H_F_), 3.67–3.56 (m, 2H, H_11_), 3.56-3.48 (m, 2H, H_20_), 2.41 (t, 2H, ^3^*J_H15-H16_* = 8 Hz, H_15_), 1.62–1.52 (m, 2H, H_19_), 1.38–1.29 (m, 2H, H_16_), 1.27 (s, 18H, H_27_), 1.01–0.93 (m, 2H, H_17_), 0.87–0.80 (m, 2H, H_18_). ^13^C-NMR (150 MHz, 5.10^−2^ M in CD_2_Cl_2_, 298K): δ (ppm) = 172.4 & 171.2 & 169.1 (4s, C_K_ C_3_ C_6_ C_9_), 160.1 & 149.1 & 135.7 (C_IV arom BMP25C8_), 154.2 (C_24_), 147.8 (C_22_), 124.9 (C_25_), 123.9 (C_13_), 122.8 (C_A_), 117.3 (C_23_), 113.2 (C_B_), 108.2 (C_I_), 106.8 (C_J_), 71.9 & 71.3 (C_E_ C_F_), 70.7 & 70.6 (C_D_ C_G_), 69.1 & 68.6 (C_C_ C_H_), 52.3 (C_20_), 50.2 (C_12_), 45.3 (C_2_), 44.2 & 44.0 (C_5_ C_8_), 39.9 (C_11_), 35.7 (C_26_), 31.6 (C_27_), 30.2 (C_16_), 29.4 (C_17_), 29.3 (C_19_), 26.2 (C_18_), 25.4 (C_15_).

*[1]rotaxane* (**13**) A solution of compound **12** (162 mg, 0.14 mmol, 1 equiv) dissolved in CH_2_Cl_2_ (10 mL) was added dropwise over 24 h to a solution of benzyl bromide (55 mL) and CH_2_Cl_2_ (218 mL). The mixture was stirred for further 6d at RT. CH_2_Cl_2_ was then evaporated and the excess of benzyl bromide was removed by filtration on a silicagel column (solvent gradient elution CH_2_Cl_2_ to CH_2_Cl_2_/MeOH 80/20). The remaining solid (224 mg) was then diluted in CH_2_Cl_2_ (16 mL). To this solution was added NH_4_PF_6_ (133 mg, 0.82 mmol, 5 equiv) and H_2_O milliQ (8 mL): the biphasic solution was stirred vigorously for 30 minutes. The aqueous layer was extracted with CH_2_Cl_2_ (3 × 20 mL) and the combined organic layers were dried over MgSO_4_ and concentrated under vacuum*.* The crude was purified twice by chromatography on a silicagel column (CH_2_Cl_2_ to CH_2_Cl_2_/MeOH 80/20) to give several mixed fractions and the isolated [1]rotaxane **13** (7 mg, 4%) as a colourless oil. R*_f_* 0.86 (CH_2_Cl_2_/MeOH 9/1). ^1^H-NMR (600 MHz, CD_2_Cl_2_, 298K): δ (ppm) = 8.60 (br s, 2H, H_21_), 8.16 (s, 1H, H_13_), 7.56 (br t, 1H, H_1_), 7.52 (s, 1H, H_25_), 7.41 (s, 2H, H_23_), 7.41–7.33 (m, 3H, H_32_ H_31_), 7.27 (d, 2H, ^3^*J_H30-H31_* = 7 Hz, H_30_), 7.27–7.19 (m, 2H, H_4_ H_7_), 7.03 (br t, 1H, H_10_), 7.01 (s, 2H, H_J_), 6.94–6.88 (m, 2H, H_A_), 6.88–6.83 (m, 2H, H_B_), 6.20 (s, 1H, H_I_), 5.64 (s, 2H, H_28_), 4.70–4.63 (m, 2H, H_12_), 4.24–4.18 & 4.18–4.12 & 4.09–4.03 (m, 8H, H_H_ H_C_), 4.01–3.95 (m, 2H, H_2_), 3.92–3.73 (m, 12H, H_5_ H_8_ H_D_ H_G_), 3.78–3.71 (m, 2H, H_11_), 3.73–3.41 (m, 8H, H_E_ H_F_), 3.51–3.41 (m, 2H, H_20_), 2.51(t, 2H, ^3^*J_H15-H16_* = 7.5 Hz, H_15_), 1.48–1.41 (m, 2H, H_19_), 1.31–1.21 (m, 2H, H_16_), 1.29 (s, 18H, H_27_), 0.88–0.80 (m, 2H, H_17_), 0.75–0.67 (m, 2H, H_18_). HSQC ^13^C-NMR (150 MHz, CD_2_Cl_2_, 298K): δ (ppm) = 129.9 (C_31_ C_32_), 129.6 (C_13_), 128.5 (C_30_), 124.7 (C_25_), 122.5 (C_A_), 117.3 (C_23_), 113.1 (C_B_), 108.8 (C_I_), 106.4 (C_J_), 72.0 & 71.2 (C_E_ C_F_), 70.6 & 69.7 (C_D_ C_G_), 68.8 & 68.5 (C_H_ C_C_), 55.2 (C_28_), 53.8 (C_12_), 52.3 (C_20_), 44.9 (C_2_), 43.5 (C_5_ C_8_), 39.3 (C_11_), 31.8 (C_27_), 28.8 (C_17_), 27.6 (C_19_), 27.1 (C_16_), 25.8 (C_18_), 23.8 (C_15_). HRMS (ESI): [M-PF_6_]^+^ calcd for C_62_H_88_F_6_N_8_O_12_P: 1281.6164, found: 1281.6177.

*[1]Rotaxane* (**14**). To a solution of the [1]rotaxane **13 **(3 mg, 2.1 µmol, 1 equiv) in CD_2_Cl_2_ (0.6 mL) was added 74 µL of a 1% solution of DIEA in CD_2_Cl_2_ (4.2 µmol, 2 equiv.). R*_f_* 0.73(CH_2_Cl_2_/MeOH 9/1). ^1^H-NMR (600 MHz, CD_2_Cl_2_, 298K): δ (ppm) = 8.29 (s, 1H, H_13_), 7.33–7.28 (m, 1H, H_32_), 7.28–7.24 (m, 2H, H_31_), 7.20 (br t, 1H, H_1_), 7.12 & 7.08 (2 br t, 2H, H_4_ H_7_), 7.03 (d, 2H, ^3^*J_H30-H31_* = 7.4 Hz, H_30_), 6.93 (br t, 1H, H_10_), 6.88–6.84 (m, 2H, H_A_), 6.83 (s, 2H, H_J_), 6.76 (s, 1H, H_25_), 6.73–6.68 (m, 2H, H_B_), 6.51 (s, 1H, H_I_), 6.48 (m, 2H, H_23_), 5.41 (s, 2H, H_28_), 4.26 (t, 2H, ^3^*J_H12-H11_* = 6.3 Hz, H_12_), 4.19–4.14 & 4.10–4.04 (m, 4H, H_H_), 4.03–3.97 & 3.94–3.89 (m, 4H, H_C_), 4.00 (d, 2H, ^3^*J_H2-H1_* = 5.3 Hz, H_2_), 3.86–3.66 (m, 12H, H_5_ H_8_ H_D_ H_G_), 3.71–3.55 (m, 8H, H_E_ H_F_), 3.55–3.50 (m, 2H, H_11_), 3.15 (t, 2H, ^3^*J_H20-H19_* = 6.5 Hz, H_20_), 3.07-3.01 (m, 2H, H_15_), 1.95–1.82 (m, 2H, H_16_), 1.69–1.62 (m, 2H, H_19_), 1.60–1.54 (m, 2H, H_17_), 1.56–1.49 (m, 2H, H_18_), 1.28 (s, 18H, H_27_). HSQC ^13^C-NMR (150 MHz, CD_2_Cl_2_, 298K): δ (ppm) = 129.4 (C_31_ C_32_), 129.3 (C_13_), 129.1 (C_30_), 121.9 (C_A_), 113.8 (C_B_), 112.1 (C_25_), 108.3 (C_I_), 107.8 (C_23_), 105.1 (C_J_), 71.8 & 71.5 & 69.9 (C_E_ C_F_), 70.9 & 70.8 & 68.4 (C_D_ C_G_), 69.8 & 69.7(C_C_), 68.6 & 68.5 (C_H_), 54.3 (C_28_), 52.3 (C_12_), 44.7 (C_20_), 44.4 (C_2_), 44.6 & 43.4 (C_5_ C_8_), 38.5 (C_11_), 31.1 (C_27_), 30.5 (C_19_), 29.9 (C_17_), 27.8 (C_18_), 26.6 (C_16_), 23.3 (C_15_). HRMS (ESI): [M+H]^+^ calcd for C_62_H_88_F_6_N_8_O_12_P: 1281.6164, found: 1281.6144.

### 3.3. Synthesis and Characterizations of the Uncomplexed Threads **13u** and **14u**

*Protonated Unthreaded Compound* (**13u)**: Compound **11** (110 mg, 0.096 mmol, 1 equiv) was dissolved in benzyl bromide (3.4 mL, 300 equiv) and the mixture was stirred for 6d at RT before being directly filtered on a silicagel column to remove the excess of benzyl bromide (solvent gradient elution CH_2_Cl_2_ to CH_2_Cl_2_/MeOH 85/15). To a solution of the residue in CH_2_Cl_2_ (4 mL) was then added TFA (1 mL). The mixture was stirred for 30 minutes before being concentrated and co-evaporated 5 times with 20 mL of CH_2_Cl_2_ in order to remove the excess of TFA. The residue was then diluted in CH_2_Cl_2_ (20 mL). To this solution was added NH_4_PF_6_ (78 mg, 0.48 mmol, 5 equiv) and MilliQ H_2_O (10 mL): the biphasic solution was stirred vigorously for 30 minutes. The aqueous layer was extracted with CH_2_Cl_2_ (3 × 5 mL) and the combined organic layers were dried over MgSO_4_ and concentrated to give compound **13u** (106 mg, 80%) as a white foam. R*_f_* 0.86 (CH_2_Cl_2_/MeOH 9/1). ^1^H-NMR (600 MHz, CD_2_Cl_2_, 298K): δ (ppm) = 8.33 (s, 1H, H_13_), 7.67 (br t, 1H, H_1_), 7.48 (br t, 1H, H_25_), 7.43 & 7.28 (2 br t, 2H, H_4_ H_7_), 7.42–7.39 (m, 3H, H_31_ H_32_), 7.30–7.26 (m, 2H, H_30_), 7.25 (d, 2H, ^4^*J_H23-H25_* = 1.4 Hz, H_23_), 7.04 (br t, 1H, H_10_), 6.99 (d, 2H, ^4^*J_HJ-HI_* = 2.1 Hz, H_J_), 6.90–6.86 (m, 5H, H_A_ H_B_ H_I_), 5.64 (s, 2H, H_28_), 4.67 (t, 2H, ^3^*J_H12-H11_* = 5 Hz, H_12_), 4.19–4.14 (m, 4H, H_H_), 4.11–4.06 (m, 4H, H_C_), 4.04 (d, 2H, ^3^*J_H2-H1_* = 5.7 Hz, H_2_), 3.88 (d, 2H, ^3^*J* = 5.7 Hz, H_5_ or H_8_), 3.83 (d, 2H, ^3^*J* = 5.6 Hz, H_5_ or H_8_), 3.81–3.75 (m, 8H, H_D_ H_G_), 3.76–3.73 (m, 2H, H_11_), 3.67 (s, 8H, H_E_ H_F_), 3.31 (t, 2H, ^3^*J_H20-H19_* = 7.8 Hz 2H, H_20_), 2.66 (t, 2H, ^3^*J_H15-H16_* = 7.4 Hz, H_15_), 1.78–1.70 (m, 2H, H_19_), 1.69–1.62 (m, 2H, H_16_), 1.38–1.30 (m, 4H, H_17_ H_18_), 1.28 (s, 18H, H_27_). ^13^C-NMR (150 MHz, CD_2_Cl_2_, 298K): δ (ppm) = 171.9 & 171.2 & 171.0 & 169.4 (C_K_ C_3_ C_6_ C_9_), 160.8 & 149.2 & 135.7 & 135.5 (C_IV arom BMP25C8_), 154.3 (C_24_), 145.1 (C_22_), 131.8 (C_29_), 130.0 (C_13_ C_31_ C_32_), 128.8 (C_30_), 124.3 (C_25_), 122.4 (C_B_), 117.2 (C_23_), 115.4 (C_A_), 107.4 (C_J_), 107.3 (C_I_), 71.1 (C_E_ C_F_), 70.3 & 70.0 (C_D_ C_G_), 69.2 (C_C_), 68.9 (C_H_), 55.4 (C_28_), 54.0 (C_20_), 53.9 (C_12_), 44.5 (C_2_), 43.8 & 43.4 (C_5_ C_8_), 39.4 (C_11_), 35.6 (C_26_), 31.4 (C_27_), 26.3 (C_16_), 28.1 & 25.9 (C_17_ C_18_ C_19_), 23.6 (C_15_). HRMS (ESI): [M−PF_6_]^+^ calcd for C_62_H_88_F_6_N_8_O_12_P: 1281.6164, found: 1281.6173.

*Deprotonated Unthreaded Compound* (**14u)**: A solution of the thread **13u** (48 mg, 0.034 mmol, 1 equiv) in CH_2_Cl_2_ (10 mL) was washed with an aqueous solution of 1 M NaOH (5 mL). After separation and further extraction of the remaining aqueous layer with CH_2_Cl_2_ (10 mL), the combined organic layer were dried over MgSO_4_ and then evaporated to obtain the deprotonated unthreaded compound **14u **(43 mg, 99%) as a colourless oil. R*_f_* 0.73 (CH_2_Cl_2_/MeOH 9/1). ^1^H-NMR (600 MHz, CD_2_Cl_2_, 298K): δ (ppm) = 8.25 (s, 1H, H_13_), 7.47 (t, 1H, ^3^*J_H1-H2_* = 6 Hz, H_1_), 7.44–7.40 (m, 3H, H_31_ H_32_), 7.42 (br t , 1H, H_4_ or H_7_), 7.22 (t, 1H, ^3^*J* = 5.4 Hz, H_4_ or H_7_), 7.30-7.26 (m, 2H, H_30_), 7.11 (t, 1H, ^3^*J_H10-H11_* = 5.9 Hz, H_10_), 6.98 (d, 2H, ^4^*J_HJ-HI_* = 2 Hz, H_J_), 6.91–6.87 (m, 4H, H_A_ H_B_), 6.85 (br t, 1H, H_I_), 6.75 (br t, 1H, H_25_), 6.43 (d, 2H, ^4^*J_H23-H25_* = 1 Hz, H_23_), 5.64 (s, 2H, H_28_), 4.63 (t, 2H, ^3^*J_H12-H11_* = 5 Hz, H_12_), 4.20–4.16 (m, 4H, H_H_), 4.11–4.07 (m, 4H, H_C_), 4.00 (d, 2H, ^3^*J_H2-H1_* = 5.5 Hz, H_2_), 3.83–3.74 (m, 12H, H_5_ H_8_ H_D_ H_G_), 3.71–3.65 (m, 2H, H_11_), 3.69 (s, 8H, H_E_ H_F_), 3.06 (t, 2H, ^3^*J_H20-H19_* = 7 Hz, H_20_), 2.71 (t, 2H, ^3^*J_H15-H16_* = 7.8 Hz, H_15_), 1.64–1.54 (m, 2H, H_16_), 1.58–1.49 (m, 2H, H_19_), 1.40–1.28 (m, 4H, H_17_ H_18_), 1.29 (s, 18H, H_27_). ^13^C-NMR (150 MHz, CD_2_Cl_2_, 298K): δ (ppm) = 172.1 & 171.0 & 170.8 & 168.9 (C_K_ C_3_ C_6_ C_9_), 160.8 & 149.4 & 135.7 (C_IV arom BMP25C8_), 152.2 (C_24_), 148.8 (C_14_), 145.2 (C_22_), 131.8 (C_29_), 130.2 & 130.1 & 130.0 (C_13_ C_31_ C_32_), 128.6 (C_30_), 122.2 (C_B_), 115.4 (C_A_), 112.2 (C_25_), 107.9 (C_23_), 107.3 (C_J_), 107.0 (C_I_), 71.3 (2s, C_E_ C_F_), 70.3 & 70.2 (C_D_ C_G_), 69.3 (C_C_), 68.9 (C_H_), 55.5 (C_28_), 54.1 (C_12_), 44.8 (C_2_), 44.5 (C_20_), 44.4 & 43.6 (C_5_ C_8_), 39.2 (C_11_), 35.2 (C_26_), 31.8 (C_27_), 29.9 (C_19_), 27.4 (C_16_), 29.2 & 27.1 (C_17_ C_18_), 23.9 (C_15_). HRMS (ESI): [M + H]^+^ calcd for C_62_H_88_F_6_N_8_O_12_P: 1281.6164, found: 1281.6168.

## 4. Conclusions

We have reported the synthesis of a new lasso molecular switch containing a peptide part, using a two-step sequence strategy: (1) the self-entanglement of a hermaphrodite molecule, (2) the trapping of the interlocked pseudo lasso structure with a molecular barrier. 1D and 2D ^1^H-NMR spectroscopies, as well as DT IM-MS studies provided information about the self-interlocking of the molecular lasso and about the pH-dependent molecular machinery. In the protonated state, the lasso remains loosened because the BMP25C8 resides around the best anilinium molecular site. However, deprotonation of the anilinium triggers the shuttling of the BMP25C8 towards the triazolium station, resulting in a more tightened conformation. It appears consistent to say that the peptide part of the lasso, which is located between the macrocycle and the triazolium unit (that is to say in the loop of the lasso), does not play any role in the molecular machinery: however, it undergoes a conformational change from a more extended and flexible shape at acidic pH to a more constraint bent conformation at basic pH. To the best of our knowledge, this is the first example of a pH-sensitive lasso molecular switch incorporating a peptide backbone. Further investigations towards the structure-activity relationship of such a peptide-containing molecular machine are in progress.

## Supplementary Materials

Supplementary materials can be accessed at: http://www.mdpi.com/1420-3049/18/9/11553/s1.
