# Supplementary material for: A pH-Sensitive Peptide-Containing Lasso Molecular Switch
Source: Molecules. 2013 Sep 17;18(9):11553–75. doi: 10.3390/molecules180911553 (PMC6270336; doi:10.3390/molecules180911553)

Supplementary Materials

Figure S1. Compound **8**: ^{1}H-NMR, CD_3OD, 298 K, 600 MHz.

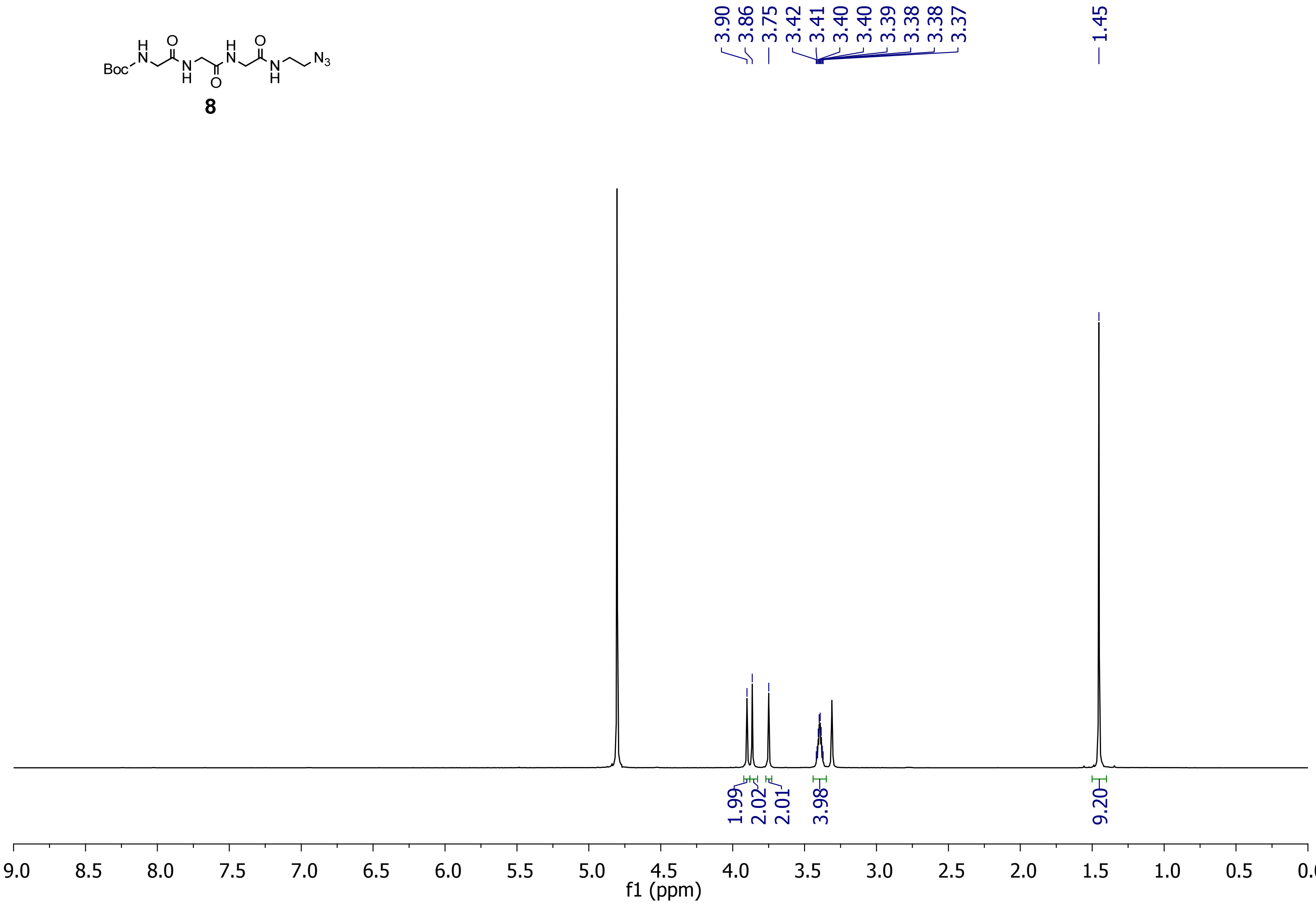

Figure S2. Compound **8**: ^{13}C-NMR, CD_3OD, 298 K, 150 MHz.

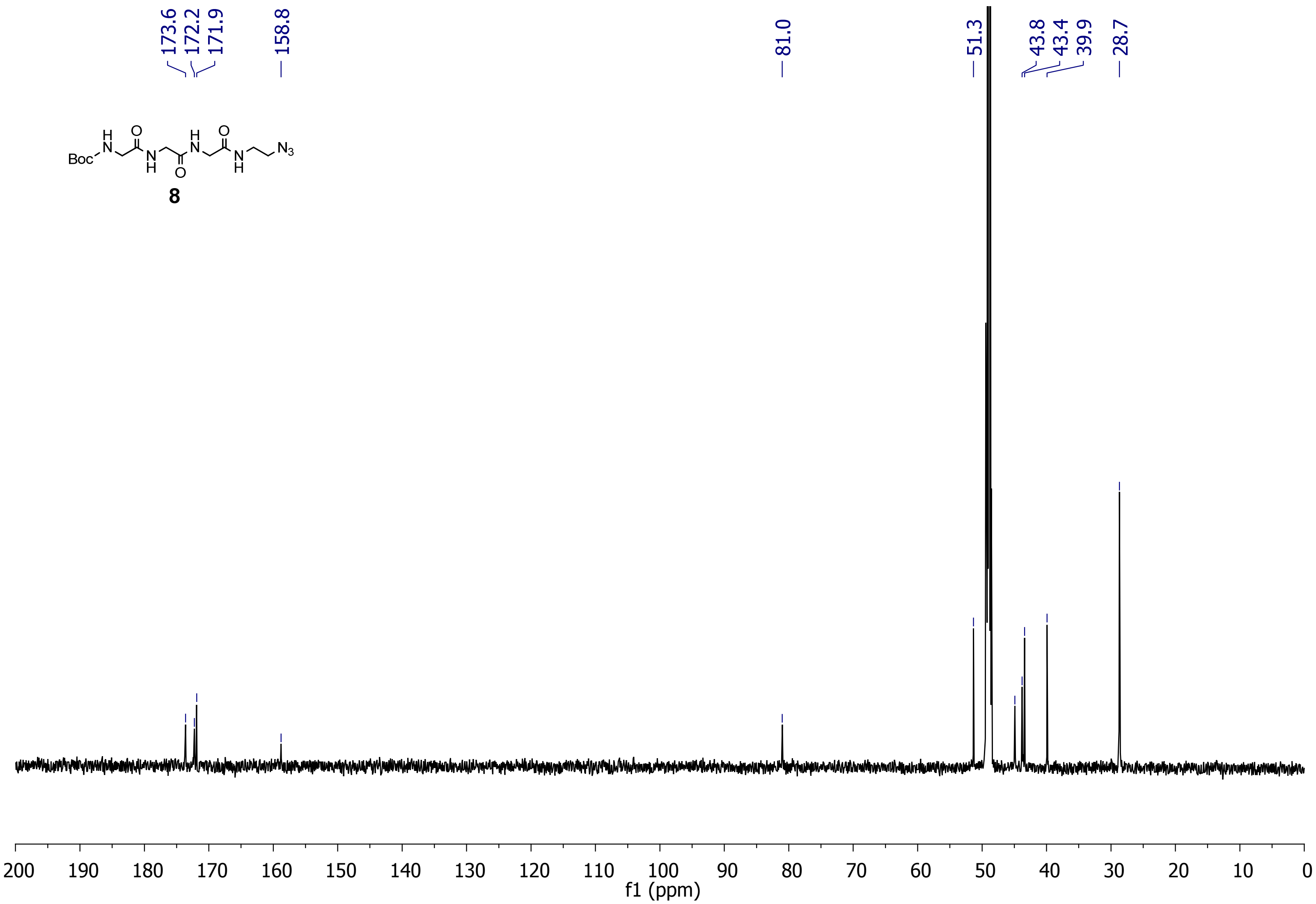

Figure S3. Compound **9**: ^{1}H-NMR, CD_3OD, 298 K, 600 MHz.

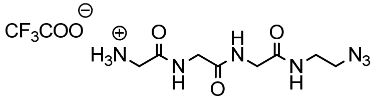

9

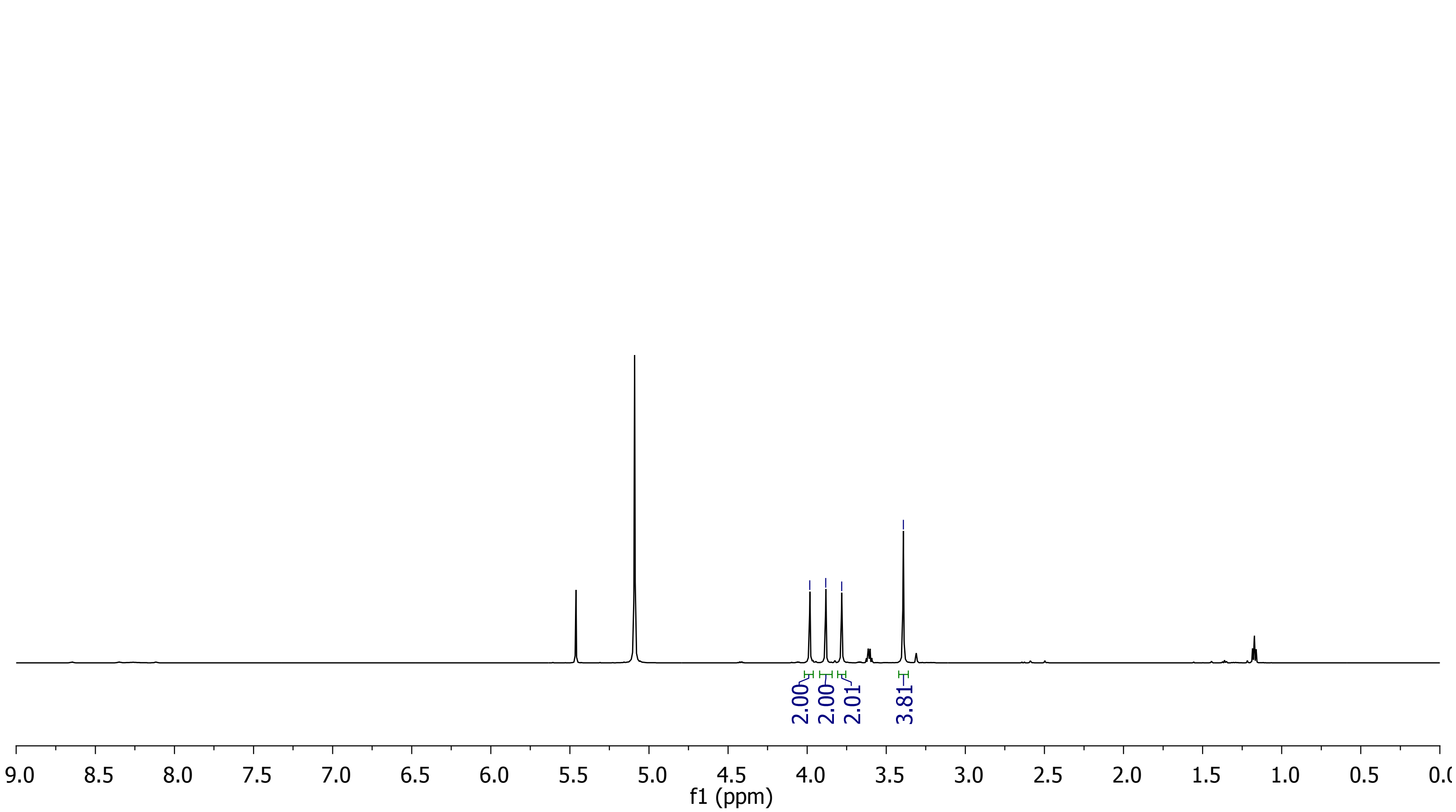

Figure S4. Compound **9**: ^{13}C-NMR, CD_3OD, 298 K, 150 MHz.

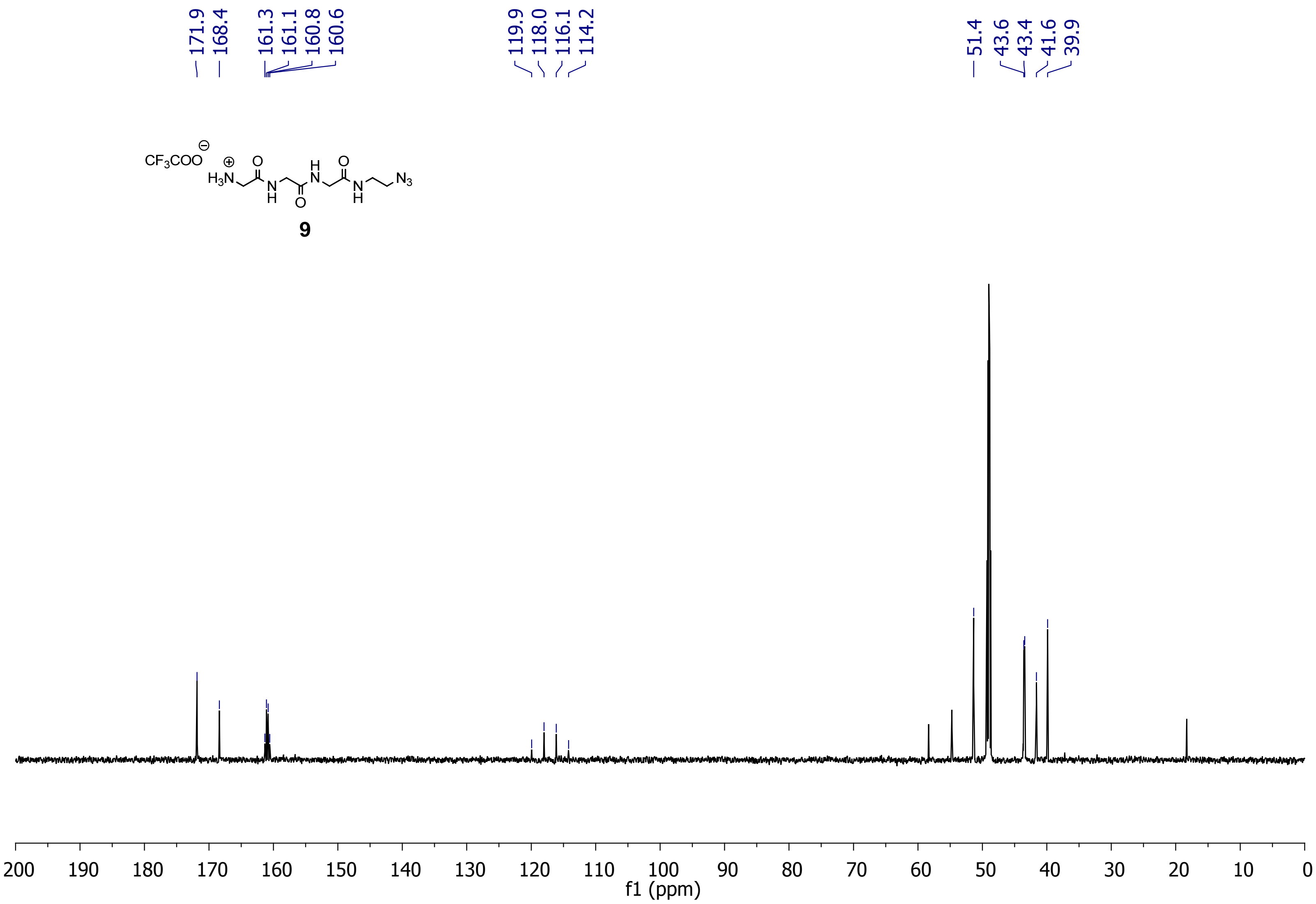

Figure S5. Compound **10**: ^{1}H-NMR, CD_3OD, 298 K, 600 MHz.

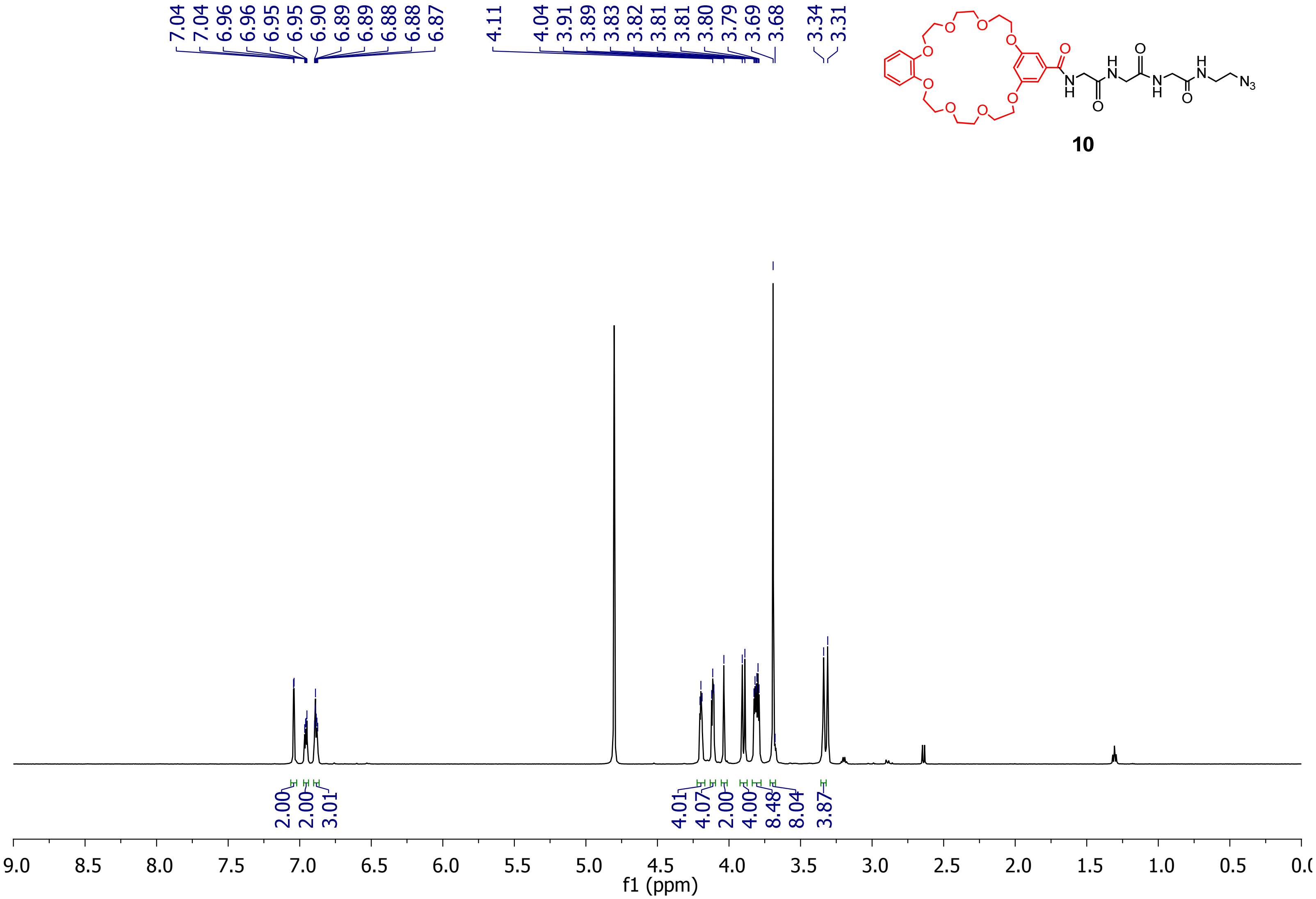

Figure S6. Compound **10**: ^{13}C-NMR, CD_3OD, 298 K, 150 MHz.

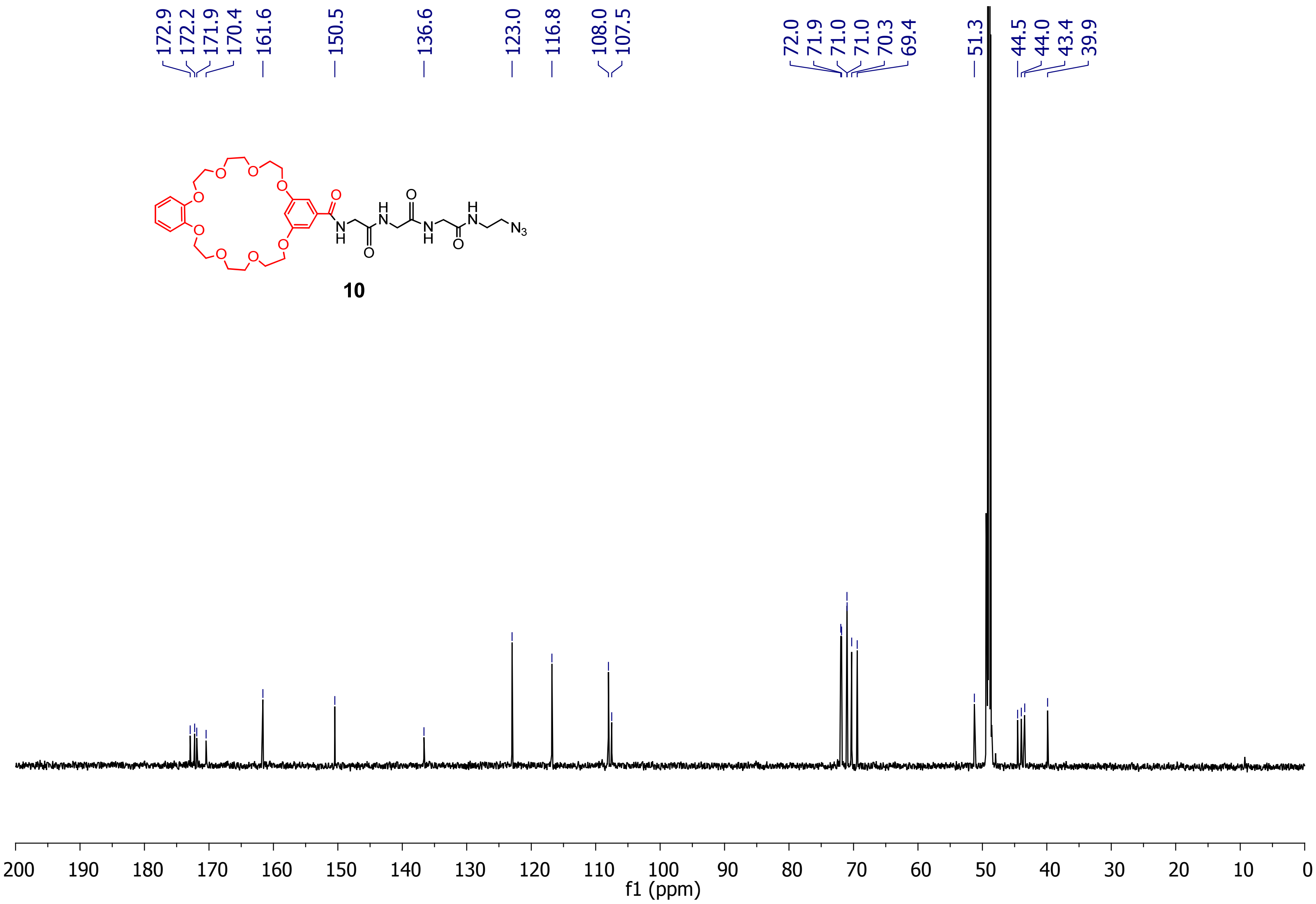

Figure S7. Compound **11**: ^{1}H-NMR, CD_2Cl_2, 298 K, 600 MHz.

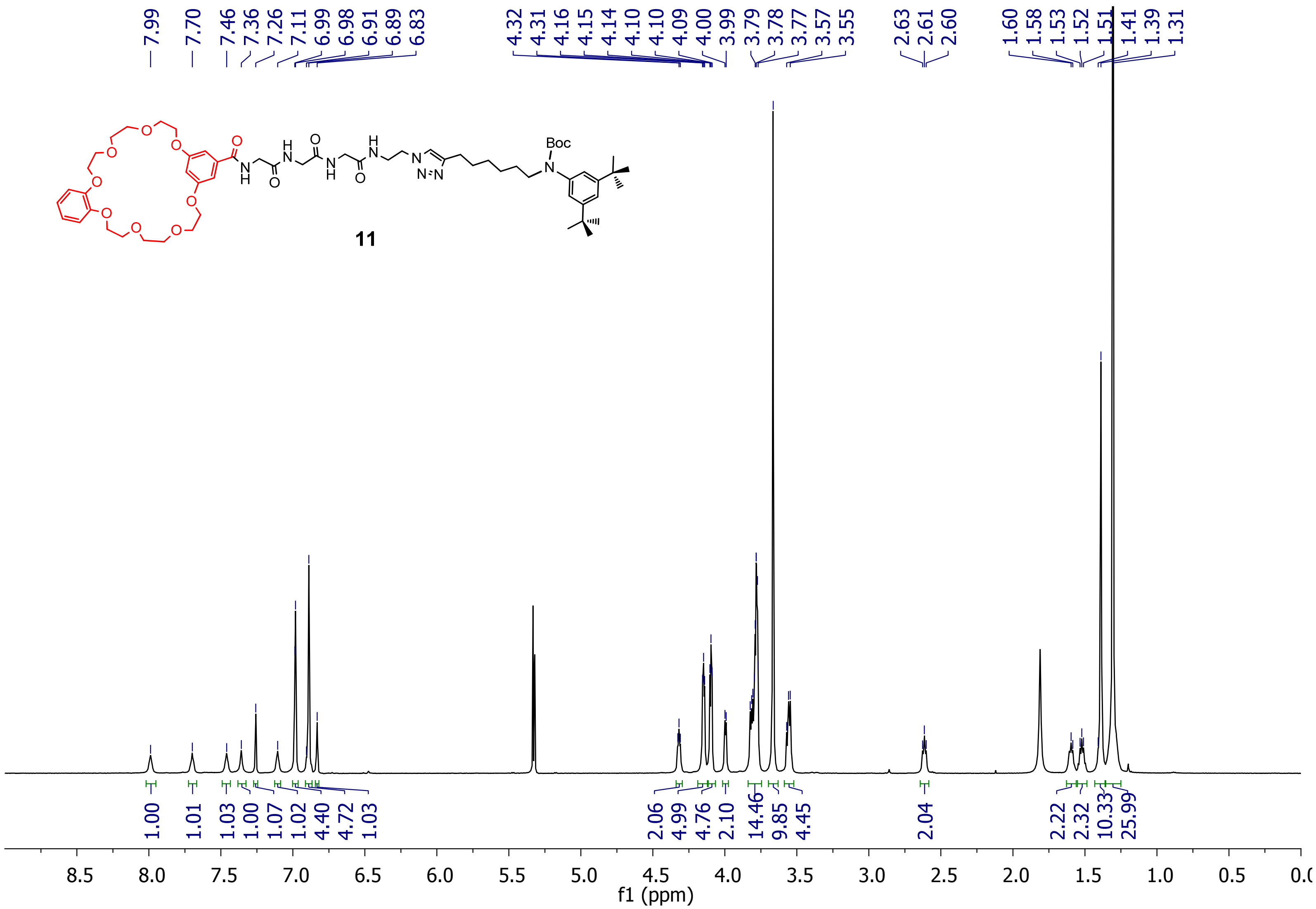

Figure S8. Compound **11**: ^{13}C-NMR, CD_2Cl_2, 298 K, 150 MHz.

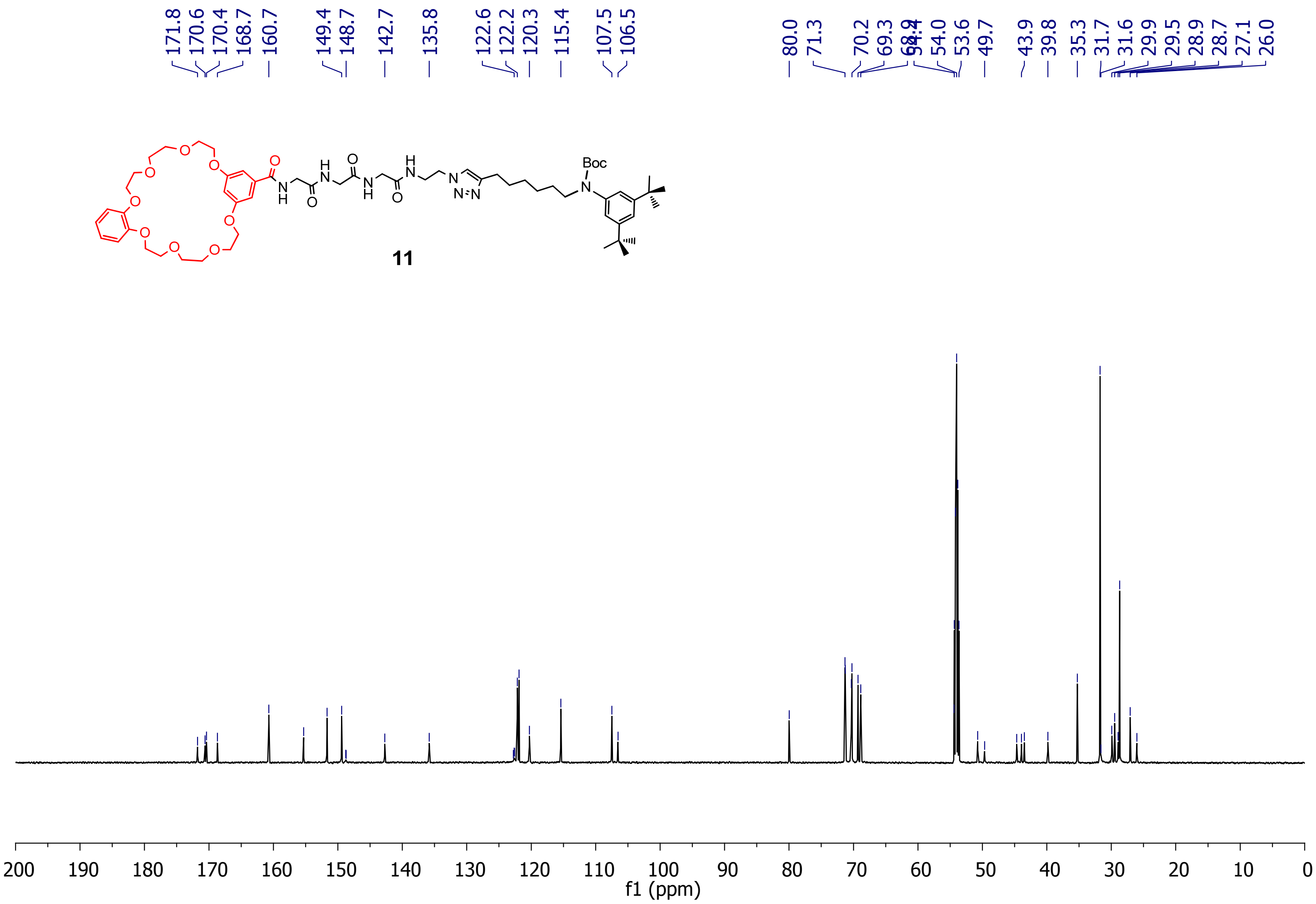

Figure S9. Compound **12+12u** (c = 5.10^{-2}M): ^{1}H-NMR, CD_2Cl_2, 298 K, 600 MHz.

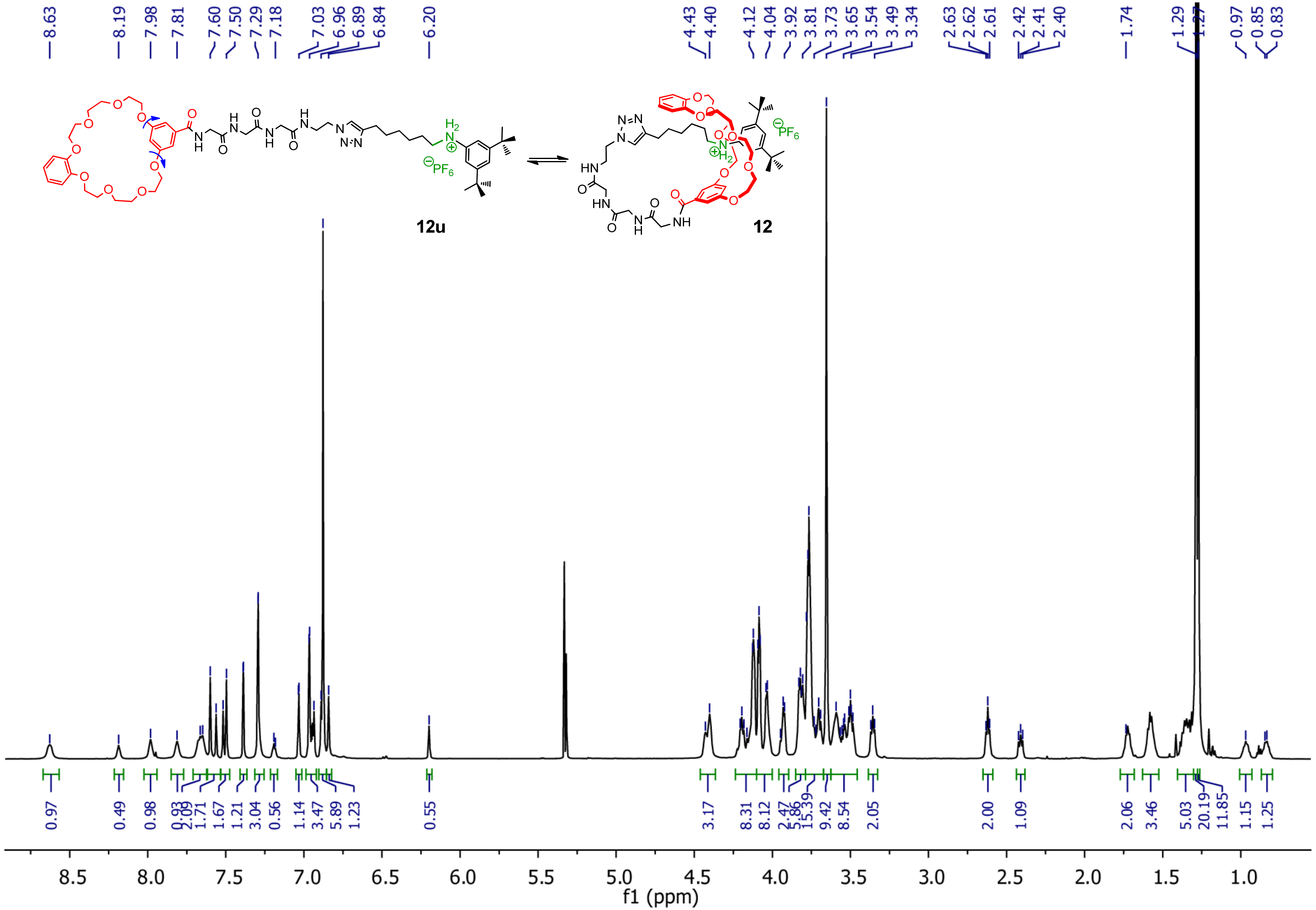

Figure S10. Compound **12+12u** (c = 5.10^{-2}M): ^{13}C-NMR, CD_2Cl_2, 298 K, 150 MHz.

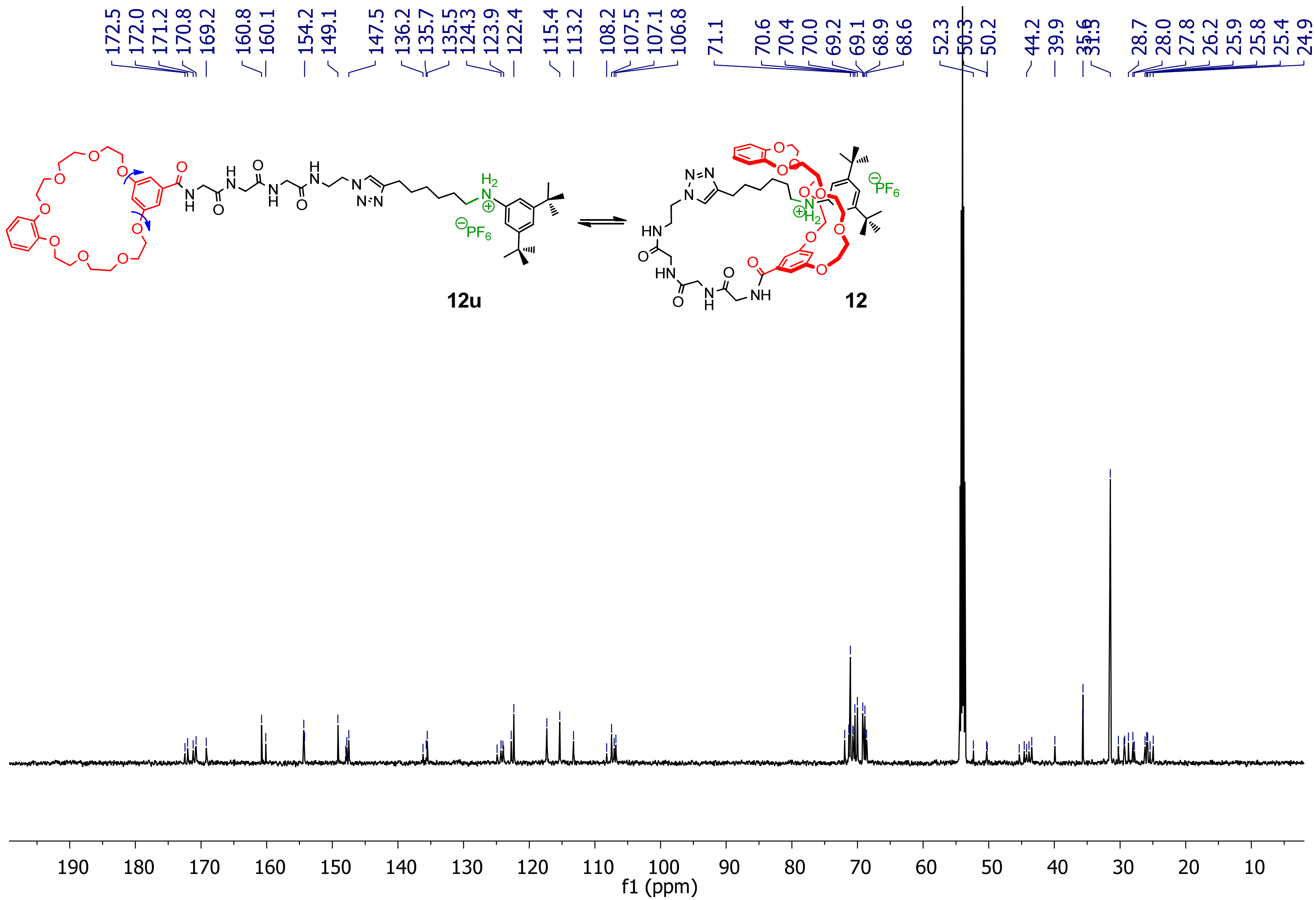

Figure S11. Compound **13**: ^{1}H-NMR, CD_2Cl_2, 298 K, 600 MHz.

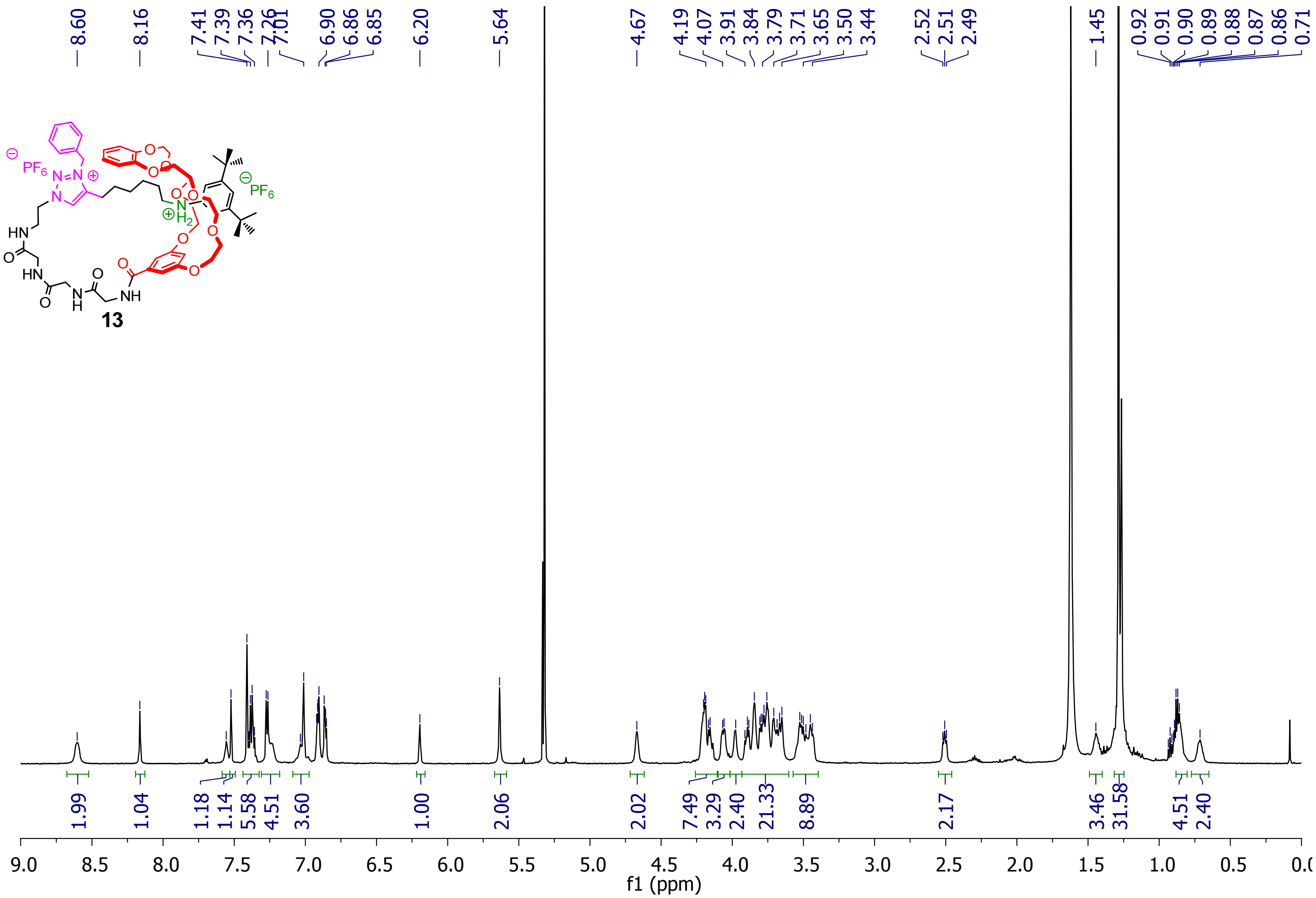

Figure S12. Compound **13**: HSQC NMR, CD_2Cl_2, 298 K, ^1H at 600MHz–^{13}C at 150 MHz

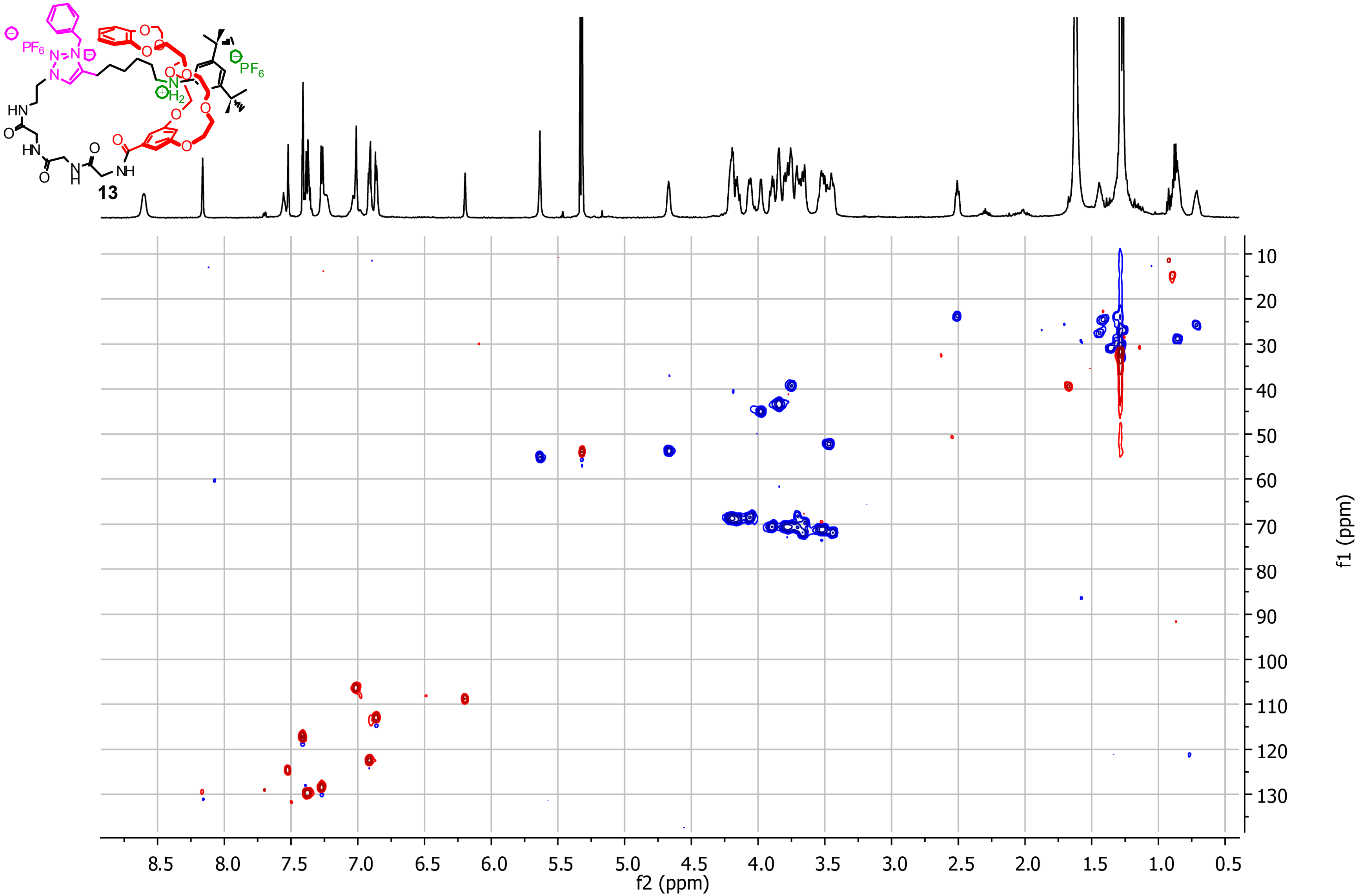

Figure S13. Compound **14**: ^{1}H-NMR, CD_2Cl_2, 298 K, 600 MHz

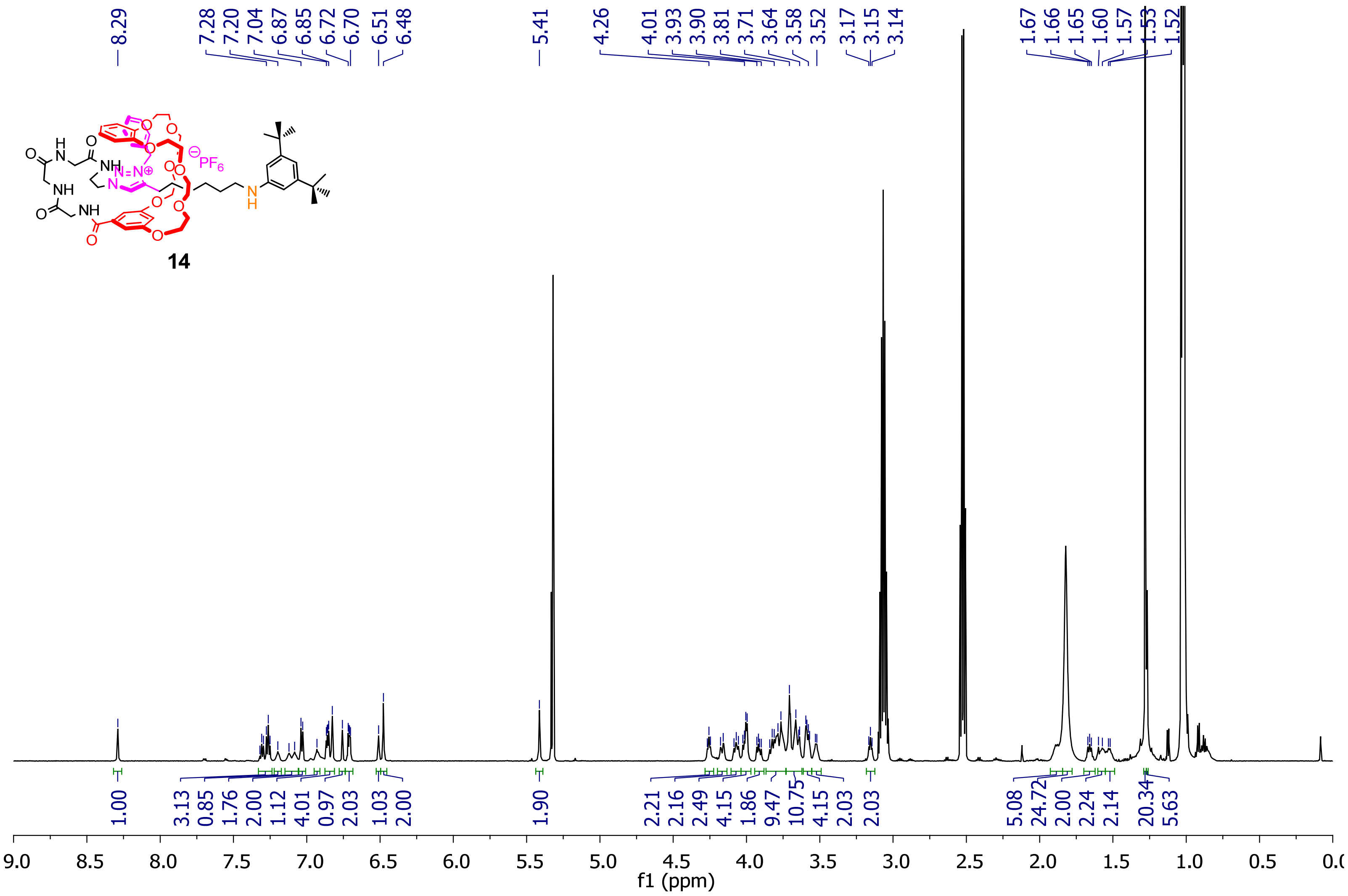

Figure S14. Compound 14: HSQC NMR, CD_2Cl_2, 298 K, ^{1}H at 600 MHz–^{13}C at 150 MHz

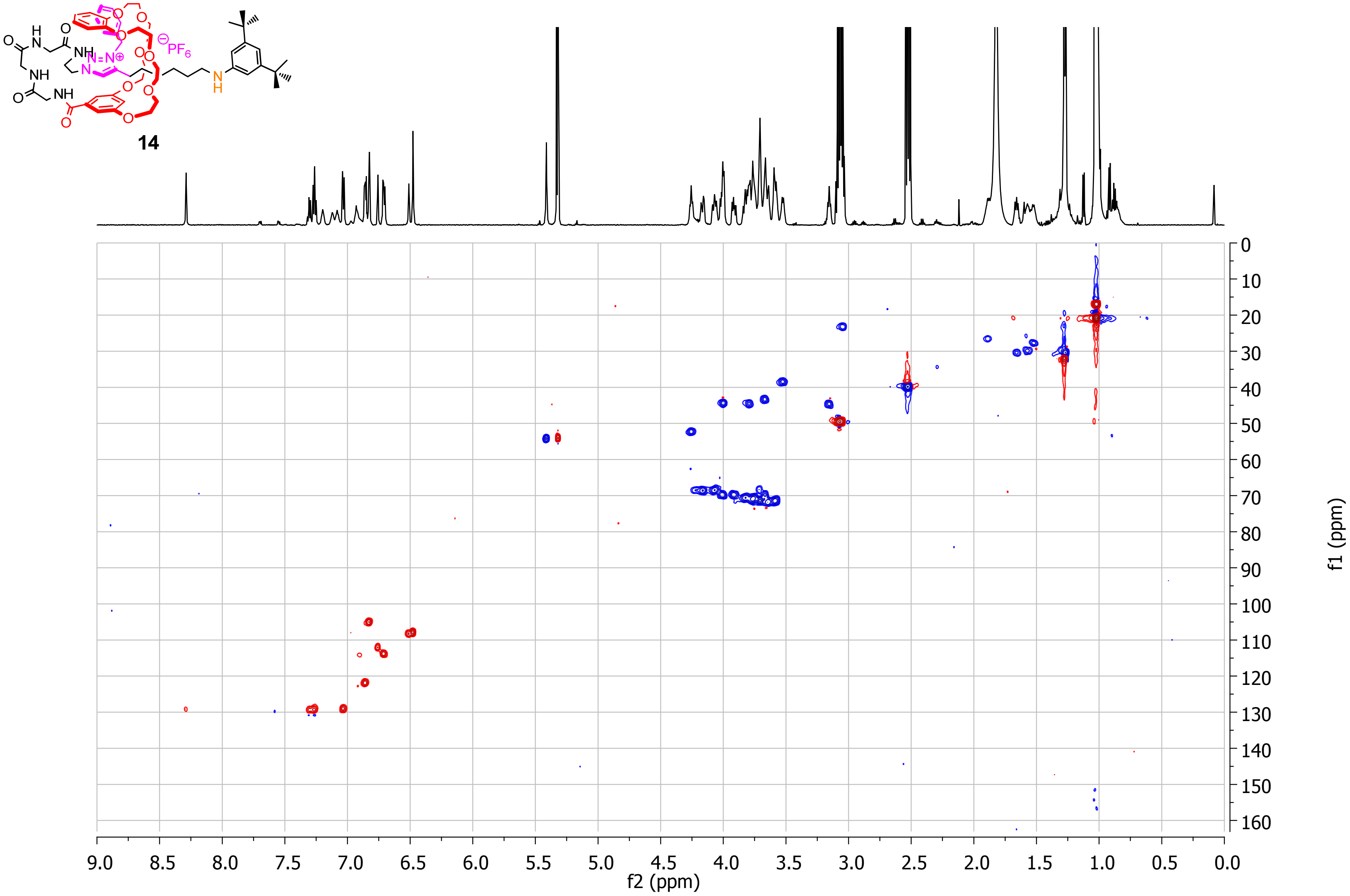

Figure S15. Compound **13u**: ^{1}H-NMR, CD_2Cl_2, 298 K, 600 MHz.

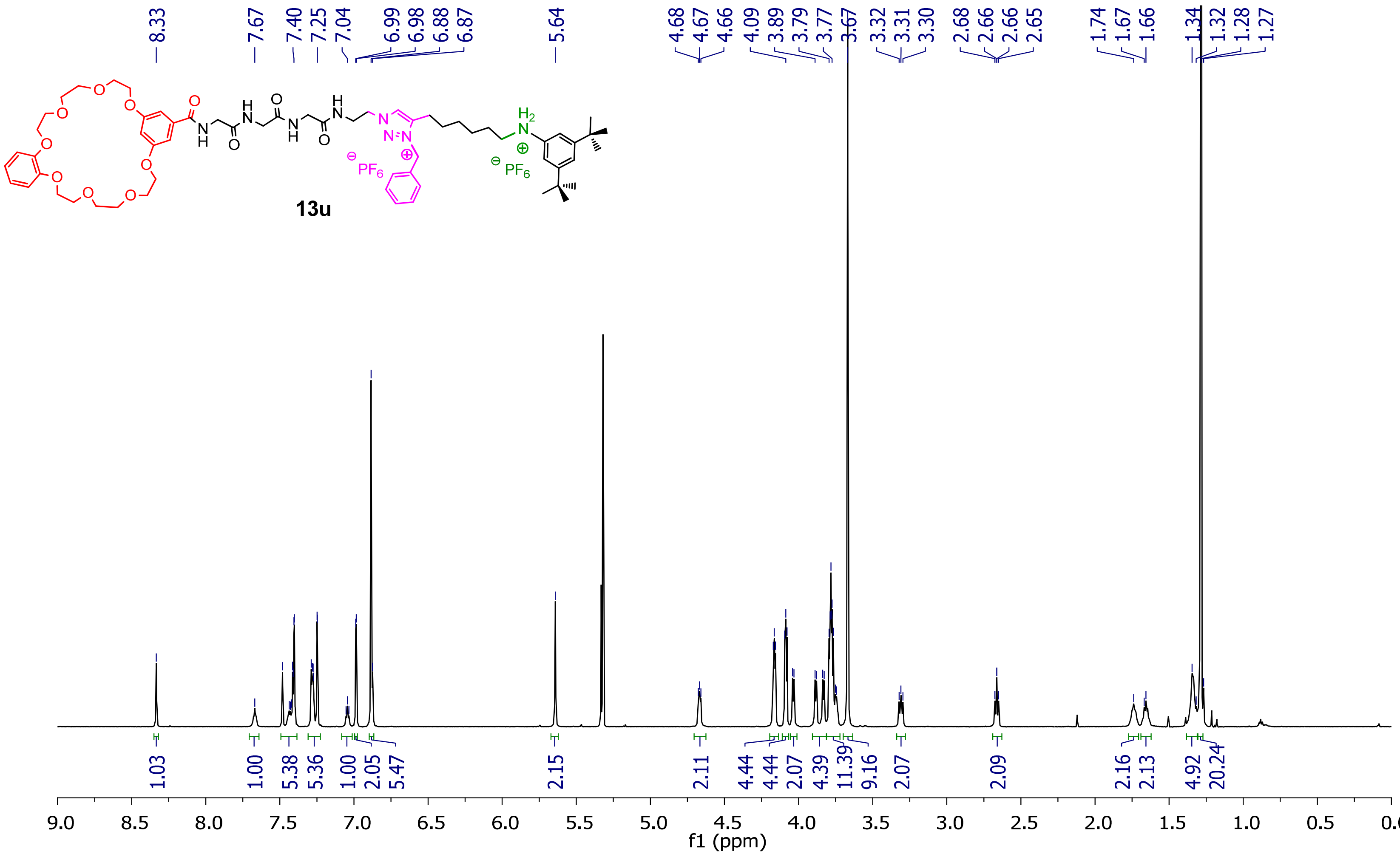

Figure S16. Compound **13u**: ^{13}C-NMR, CD_2Cl_2, 298 K, 150 MHz

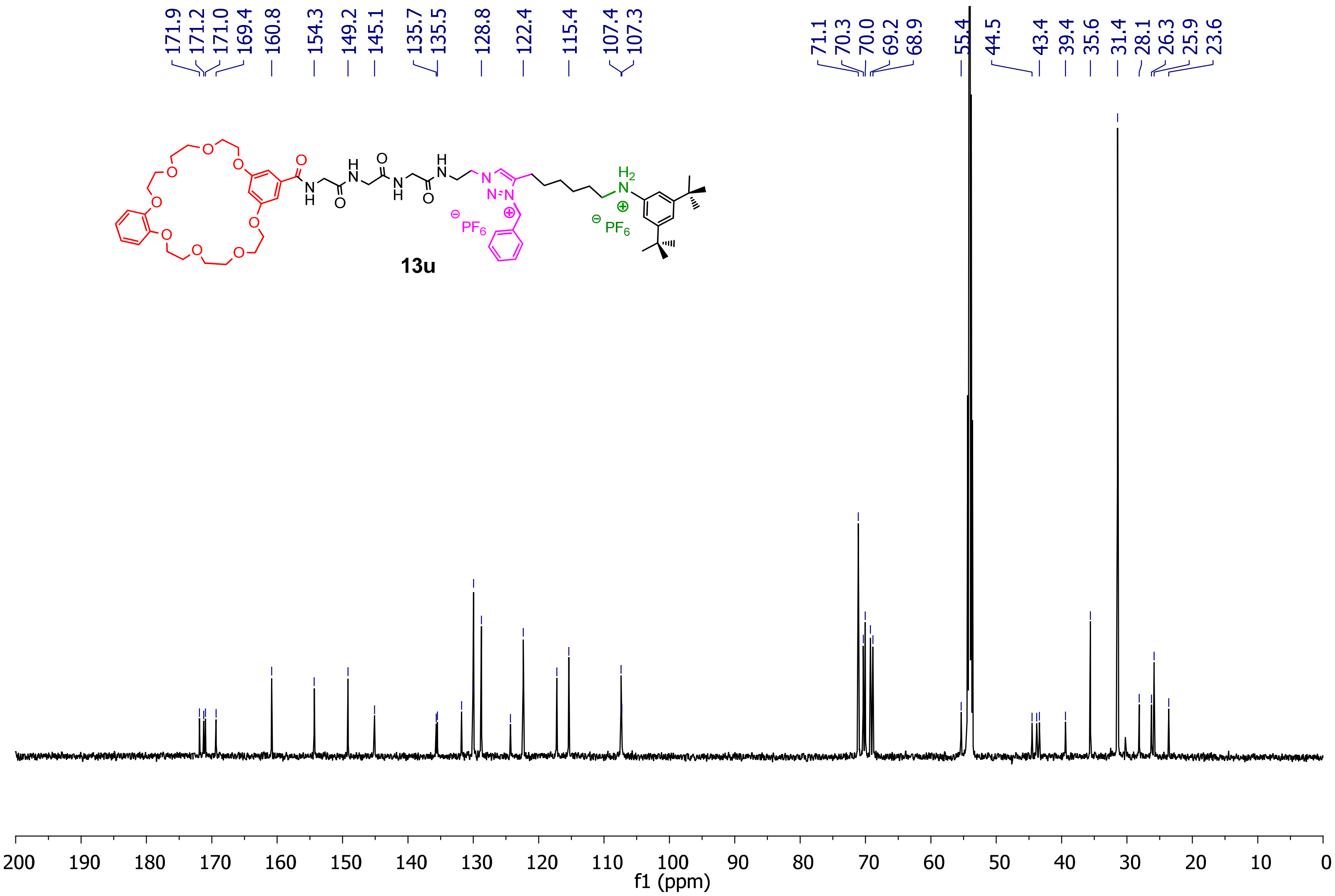

Figure S17. Compound **14u**: ^{1}H-NMR, CD_2Cl_2, 298 K, 600 MHz.

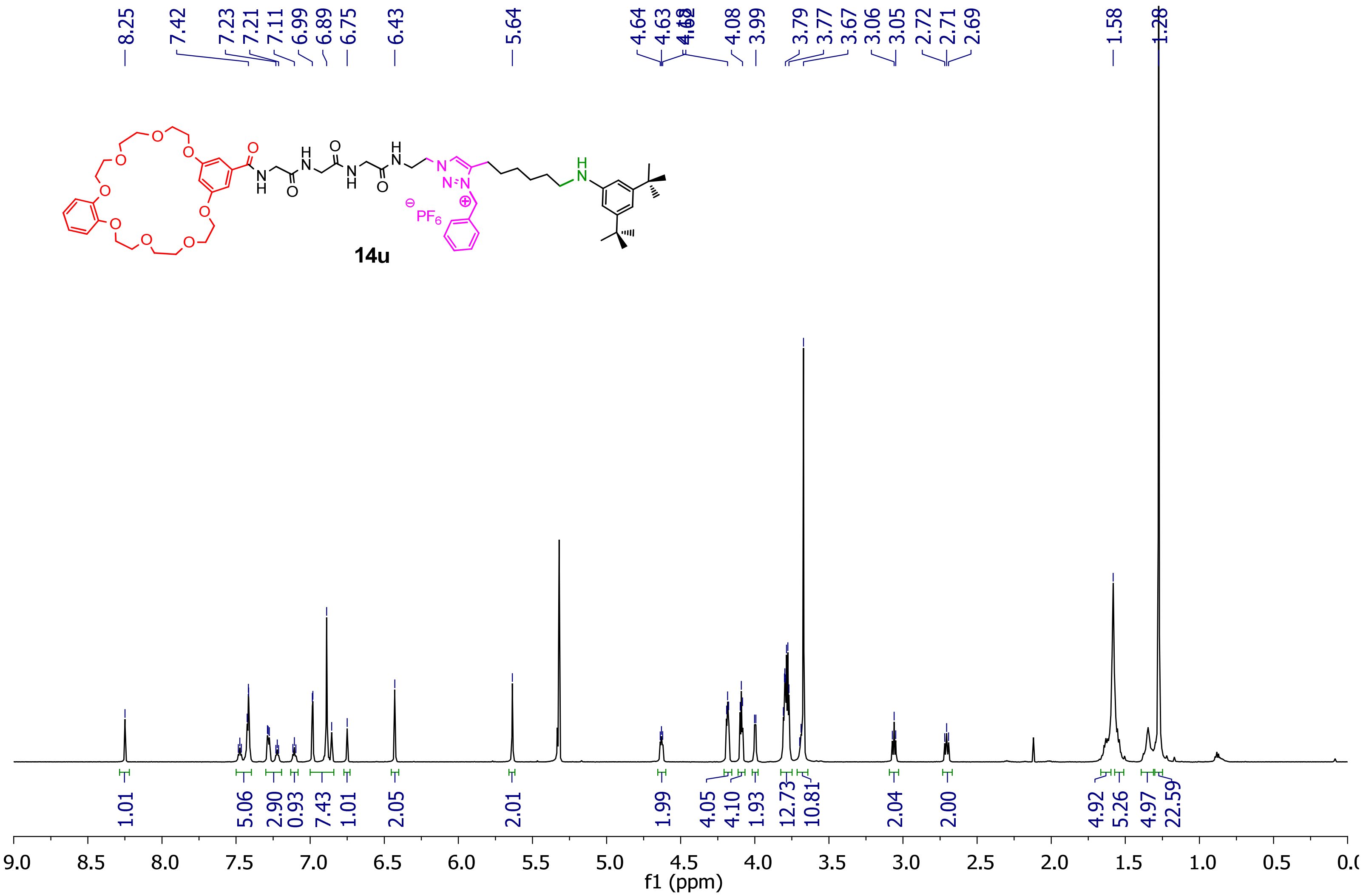

Figure S18. Compound **14u**: ^{13}C-NMR, CD_2Cl_2, 298 K, 150 MHz.

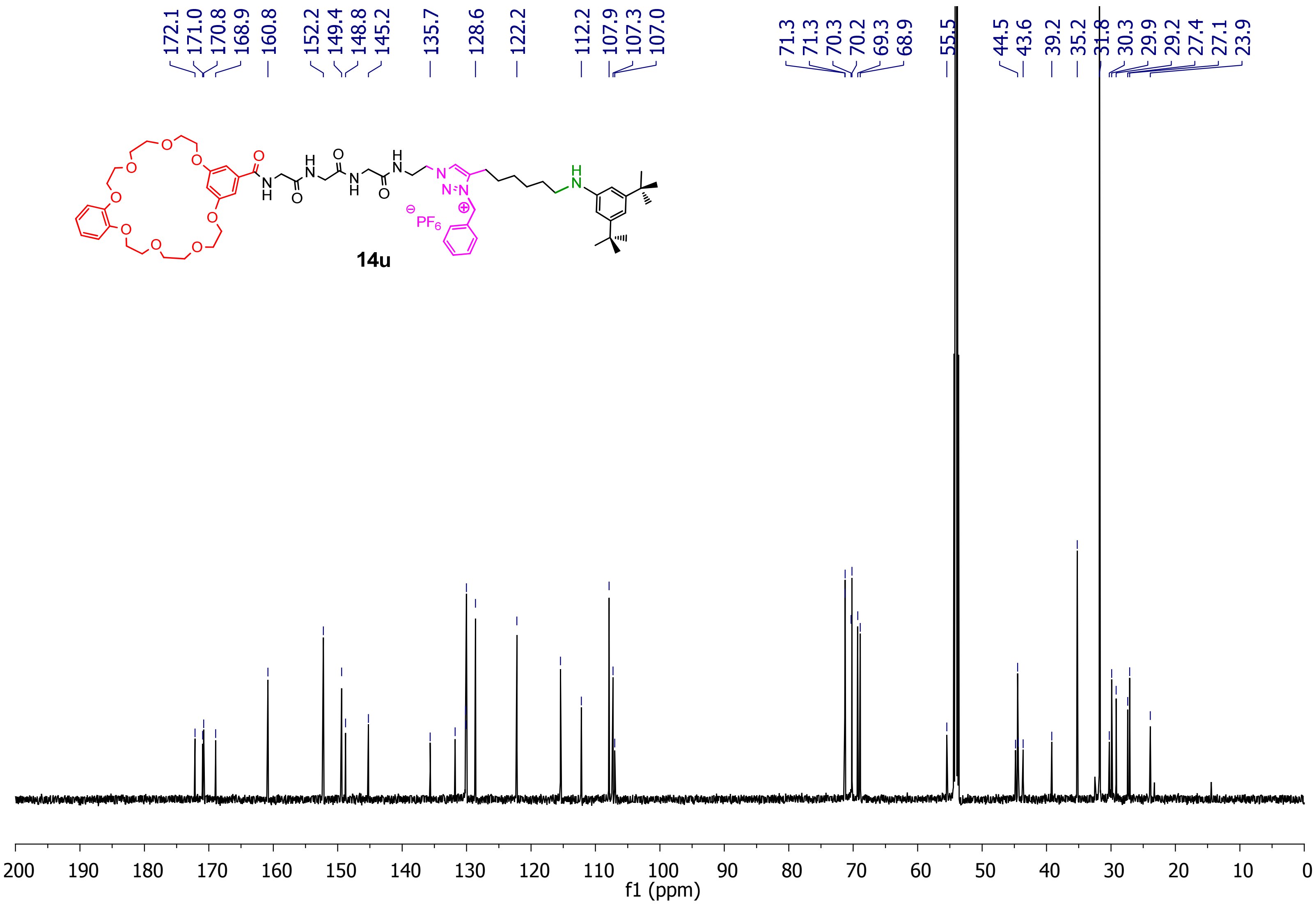

Supplement: Supplementary file 1 [file molecules-18-11553-s001.pdf]
